# An orexigenic subnetwork within the human hippocampus

**DOI:** 10.1038/s41586-023-06459-w

**Published:** 2023-08-30

**Authors:** Daniel A. N. Barbosa, Sandra Gattas, Juliana S. Salgado, Fiene Marie Kuijper, Allan R. Wang, Yuhao Huang, Bina Kakusa, Christoph Leuze, Artur Luczak, Paul Rapp, Robert C. Malenka, Dora Hermes, Kai J. Miller, Boris D. Heifets, Cara Bohon, Jennifer A. McNab, Casey H. Halpern

**Affiliations:** 1 Department of Neurosurgery, Perelman School of Medicine, University of Pennsylvania, Philadelphia, PA USA; 2 Department of Electrical Engineering and Computer Science, University of California, Irvine, Irvine, CA USA; 3 Department of Anesthesiology, Perioperative and Pain Medicine, Stanford University School of Medicine, Stanford, CA USA; 4 Department of Neurosurgery, Stanford University School of Medicine, Stanford, CA USA; 5 Université Paris Cité, Paris, France; 6 Assistance Publique des Hôpitaux de Paris, Paris, France; 7 Department of Radiology, Stanford University School of Medicine, Stanford, CA USA; 8 Canadian Centre for Behavioral Neuroscience, University of Lethbridge, Lethbridge, Alberta Canada; 9 Department of Military & Emergency Medicine, Uniformed Services University, Bethesda, MD USA; 10 Department of Psychiatry and Behavioral Sciences, Stanford University School of Medicine, Stanford, CA USA; 11 Nancy Pritzker Laboratory, Department of Psychiatry and Behavioral Sciences, Stanford University School of Medicine, Stanford, CA USA; 12 Department of Physiology & Biomedical Engineering, Mayo Clinic, Rochester, MN USA; 13 Department of Neurosurgery, Mayo Clinic, Rochester, MN USA; 14 Department of Surgery, Corporal Michael J. Crescenz Veterans Affairs Medical Center, Philadelphia, PA USA

**Keywords:** Neuroscience, Feeding behaviour, Neurophysiology, Psychiatric disorders

## Abstract

Only recently have more specific circuit-probing techniques become available to inform previous reports implicating the rodent hippocampus in orexigenic appetitive processing^1–4^. This function has been reported to be mediated at least in part by lateral hypothalamic inputs, including those involving orexigenic lateral hypothalamic neuropeptides, such as melanin-concentrating hormone^5,6^. This circuit, however, remains elusive in humans. Here we combine tractography, intracranial electrophysiology, cortico-subcortical evoked potentials, and brain-clearing 3D histology to identify an orexigenic circuit involving the lateral hypothalamus and converging in a hippocampal subregion. We found that low-frequency power is modulated by sweet-fat food cues, and this modulation was specific to the dorsolateral hippocampus. Structural and functional analyses of this circuit in a human cohort exhibiting dysregulated eating behaviour revealed connectivity that was inversely related to body mass index. Collectively, this multimodal approach describes an orexigenic subnetwork within the human hippocampus implicated in obesity and related eating disorders.

## Abstract (web summary)

An appetite-regulating subnetwork in humans involving the lateral hypothalamus and the dorsolateral hippocampus is implicated in obesity and related eating disorders.

## Main

Orexigenic appetitive processing relies on the integration of sensory, interoceptive and hormonal signals to govern consummatory behaviours^1,7^. Dysregulation of this process leads to maladaptive eating behaviour such as binge eating and is associated with obesity^8^. Studies in rodents have demonstrated that hippocampal neuronal subpopulations respond to food cues and encode food-place memory^1,2^. Projections from the lateral hypothalamus (LH) are central to this orexigenic hippocampal function, as disturbance of this circuit leads to dysregulated eating behaviour^5^. These LH projections were found to express melanin-concentrating hormone (MCH)^9^, an orexigenic neuropeptide that is produced in the LH area (refers to the LH and its adjacencies, including parts of the zona incerta)^5^. MCH-containing projection neurons have been reported to influence the reward value of food with MCH overexpression being linked with the obese state^10,11^.

The underlying circuit in which the LH and hippocampus interact, and its relevance to orexigenic appetitive processing in humans, which includes the pre-oral cue-driven process, are yet to be examined. Here we characterize the structural and functional involvement of the human hippocampus in food-related appetitive processing.

## Appetitive processing within the dlHPC

**Fig. 1:**
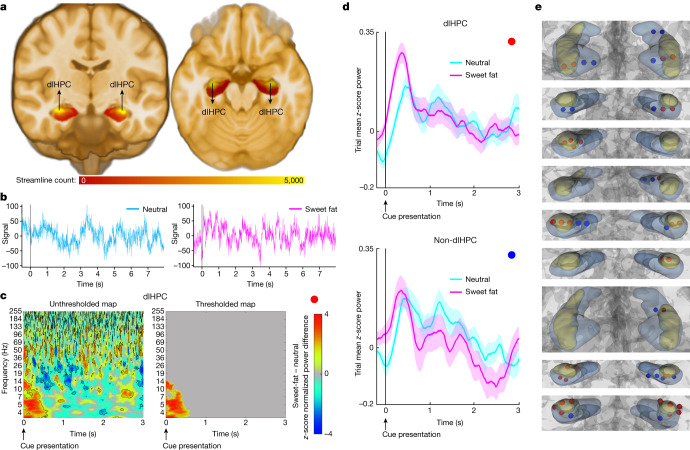
dlHPC subregion involvement in food-related appetitive processing. **a**, Tractography analysis of high-resolution, normative data from 178 participants from the HCP showing that tractography-defined LH–hippocampal area interconnections (that is, streamlines) converge in the dlHPC (yellow). **b**, Example traces of electrophysiological time-domain recordings from the dlHPC in one individual during a taste-neutral (left, cyan) and a sweet-fat (right, magenta) trial. The time interval displayed includes the pre-cue period (−0.5 to 0 s), cue presentation (0–1 s), fixation cross (1–3 s), solution delivery (3–5 s), fixation cross (5–6 s) and a portion of the remaining duration of solution receipt/consummatory phase (6–7.5 s). The detailed task paradigm is described in Supplementary Fig. 1. **c**, *z*-score-normalized difference spectrograms (sweet-fat minus taste-neutral solution) in the dlHPC. The colour bar indicates mean *z*-score power difference (using pooled channels as observations) between the two conditions compared with a null distribution. The outlined clusters (left) reflect significant contiguous time–frequency voxels (*P*  < 0.05, two-sided paired nonparametric cluster-based permutation testing, 1,000 permutations, *n* = 34 channels) before correction for multiple comparisons. The thresholded map (right) displays significant time–frequency clusters after correction for multiple comparisons using cluster size (Methods). **d**, 4–6 Hz mean z-score power time traces during cue (0–1 s) and after cue anticipation (1–3 s) of sweet-fat (magenta) and taste-neutral (cyan) solutions in the dlHPC (top) and non-dlHPC (bottom) hippocampal sites. 0 s and 1 s reflect the cue and fixation cross image presentation times, respectively. **e**, Hippocampal coverage per participant (*n* = 9). The red contacts indicate the contacts in direct contact with the dlHPC (yellow subregion). 3D volumes were rendered using DSI Studio (v.2022; publicly available at https://dsi-studio.labsolver.org/). Source Data

Using probabilistic tractography in high-resolution, normative data from the 7T Human Connectome Project (HCP) release (*n* = 178), we found that tractography-defined LH interconnections (streamlines) converge in the dorsolateral hippocampus (dlHPC) subregion (Fig. 1a). We next investigated the functional involvement of dlHPC in the processing of a palatable taste. For brevity, we refer to the volume of the hippocampus outside the dlHPC subregion as the non-dlHPC subregion. More specifically, we tested the following hypotheses: (1) dlHPC spectral dynamics discriminate between sweet-fat and neutral cues; and (2) spectral dynamics will differ between electrodes in direct contact with dlHPC and those in direct contact only with the non-dlHPC subregion. We measured local field potential activity (Fig. 1b) using intracranial electrodes (*n* = 54; 34 dlHPC contacts, 20 non-dlHPC contacts) implanted into the human hippocampus while the participants (*n* = 9) performed a sweet-fat incentive task paradigm^12^ (Supplementary Fig. 1a). The demographic and clinical characteristics of all of the participants are described in Supplementary Table 1. In this paradigm, individuals were cued for 1 s with an image representative of either a sweet-fat or taste-neutral solution to be subsequently delivered for consumption. We found that condition-specific prestimulus-normalized low-frequency power (around 3–14 Hz, with a primarily sustained peak of about 4–6 Hz; referred to hereafter as the low-frequency power cluster to reflect the frequency range of this cluster) in the dlHPC was significantly higher (*P* < 0.05, paired nonparametric cluster-based permutation testing, using null-distribution cluster size to correct for multiple comparisons) during anticipation of the sweet-fat solution compared with a neutral taste (Fig. 1c). While higher frequencies may reflect more local activity, lower frequencies are thought to be advantageous in routing information across distant areas as their longer period accommodates the temporal demand of conduction velocity across multiple synaptic delays^13^. This profile was observed immediately after the cue (around 110 ms) and was localized to contacts within the dlHPC subregion (Fig. 1d,e).

**Extended Data Fig. 1:**
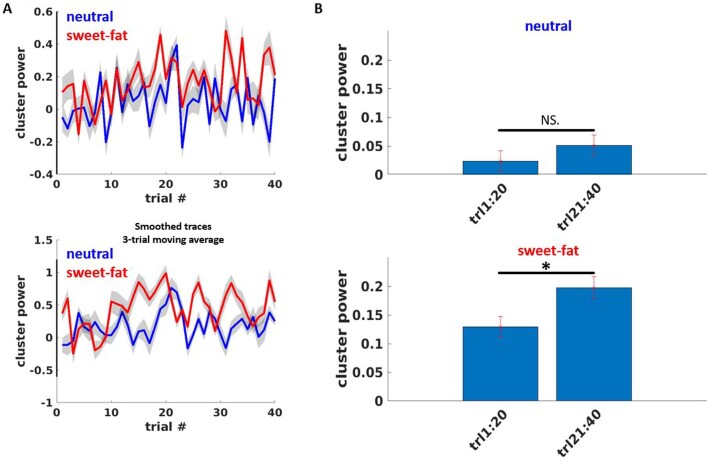
Relationship between the low frequency cluster power and trial number in the task. Trial number used as proxy measure for sweet-fat solution expectation. (A) Cluster power plotted as a function of trial number in the task (top: individual trial power, bottom: smoothed data by averaging 3 consecutive trial values). (B) Cluster power in the first versus last 20 trials of the task for the neutral (top) and sweet-fat (bottom) conditions. Note that the last 20 trials had significantly higher cluster power compared to the first 20 trials for the sweet-fat (*p* = 0.014, one-sided, unpaired permutation testing) but not for the taste-neutral condition (*p* = 0.198). NS. = non-significant, * = *p* < .05, error bars represent standard error of the mean across pooled trials.

We further investigated a potential role for the dlHPC low-frequency power cluster in learning the cue-palatable association. We hypothesized that, if cluster power encodes an association between the visual cue and the appetitive reward, then power would increase as a function of trial number. This is because the participants learn the association of the visual cue and the appetitive solution, as well as the taste of the solution as a function of trial number. Indeed, cluster power was significantly correlated with trial number for sweet-fat item anticipation (*R* = 0.102, *P* = 0.0021) but not for the taste-neutral item (*R* = 0.035, *P* = 0.292; Extended Data Fig. 1a). Moreover, the last 20 trials had significantly higher cluster power compared with the first 20 trials for the sweet-fat condition (*P* = 0.014, unpaired permutation testing) but not for the taste-neutral condition (*P* = 0.198; Extended Data Fig. 1b). These results are contrary to the low-frequency power cluster reflecting mere visual processing. Repeated presentations of a visual stimulus is thought to lead to repetition suppression, possibly reflecting recognition of old versus new items. Repetition suppression is measured as decreased hippocampal blood-oxygen-level-dependent (BOLD) activity^14,15^, and in hippocampal invasive electroencephalography (iEEG) data, it has been captured as decreased gamma and increased alpha (10–15Hz) power with repetition number, occurring 600–1,200 ms after stimulus presentation^16^. Rather, these results support the hypothesis that this early low-frequency power cluster is increased as the cue-palatable association is learned.

**Extended Data Fig. 2:**
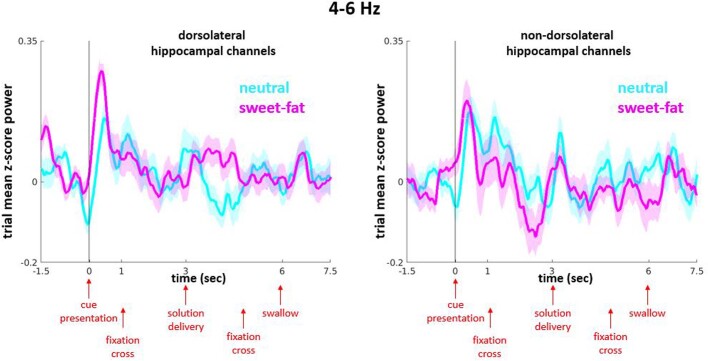
4–6 Hz power time course throughout the entirety of the trial. 4–6 Hz mean z-score power time traces in the dlHPC (left traces) and non-dlHPC (right traces) hippocampal sites, respectively. The time interval displayed includes the pre-cue period (−1.5 to 0 s), cue presentation (0-1 s), fixation cross (1–3 s), solution delivery (3–5 s), fixation cross (5-6 s), and a portion of the remaining duration of solution receipt/consummatory phase (6–7.5 s). Note increased 4–6 Hz power for sweet-fat solution is specific to the dorsolateral contacts during both cue presentation and solution delivery. Source Data

**Extended Data Fig. 3:**
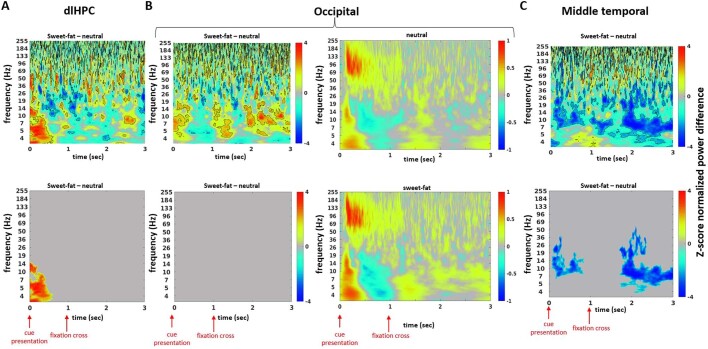
dlHPC low frequency cluster power involvement in appetitive food processing is not observed in control brain areas. (A) Z-score normalized difference spectrograms between the sweet-fat minus the taste-neutral items in the sweet-fat incentive paradigm. Note, this is a repeat of Fig. 1c included here as a reference. (B, left, C) Z-score normalized difference spectrograms between the sweet-fat minus the taste-neutral items in the sweet-fat incentive paradigm in occipital (B) and middle temporal (C) control areas. (B, right) Spectrograms for taste-neutral (top) and sweet-fat (bottom) items, displayed separately for each item.

**Extended Data Fig. 4:**
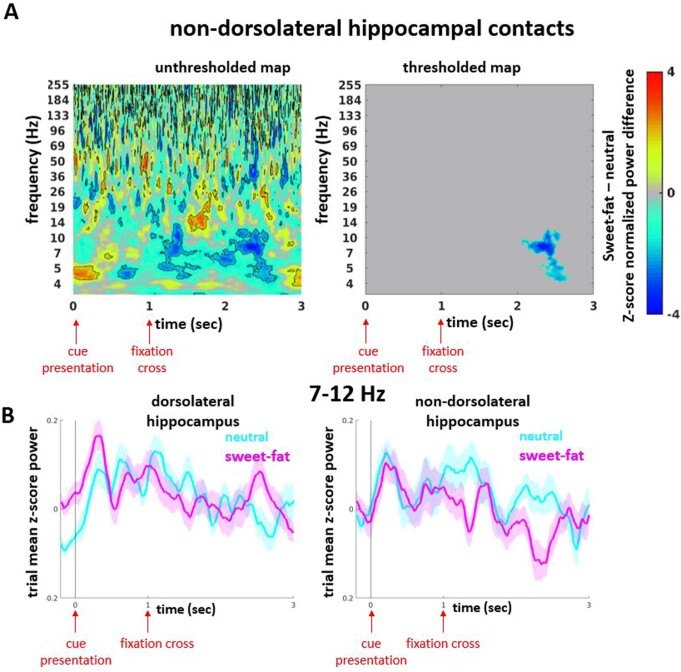
Non-dorsolateral hippocampal spectral content during food anticipation. (A) Z-score normalized difference spectrograms (sweet-fat – taste-neutral item) in the non-dorsolateral hippocampus (non-dlHPC) before (left spectrogram) and after (right spectrogram) correction for multiple comparisons. Colour bar indicates mean z-score power difference (using pooled channels as observations) between the two conditions compared to a null distribution. Outlined clusters (left spectrogram) reflect significant contiguous time-frequency voxels (p < 0.05, two-sided cluster-based permutation testing, 1000 permutations) before correction for multiple comparisons. Thresholded map displays significant time-frequency cluster (~ 7–12 Hz) after correction for multiple comparisons using cluster size (see methods). Note the absence of a cluster centred in ~4–6 Hz in the thresholded map. (B) 7–12 Hz mean z-score power time traces in the dlHPC (left traces) and non-dlHPC (right traces) hippocampal sites, respectively. 0-time indicates cue presentation. Note that while 7–12 Hz power is recruited in both subregions, condition specificity to water at this frequency range is only observed in non-dlHPC.

**Extended Data Fig. 5:**
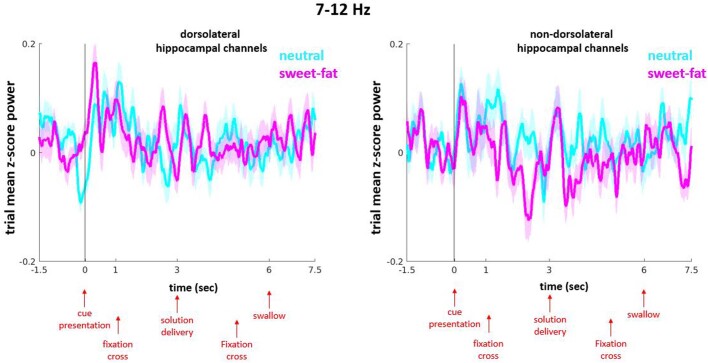
7–12 Hz power time course throughout the entirety of the trial. 7–12 Hz mean z-score power time traces in the dlHPC (left traces) and non-dlHPC (right traces) hippocampal sites, respectively. The time interval displayed includes the pre-cue period (−1.5 to 0 s), cue presentation (0-1 s), fixation cross (1–3 s), solution delivery (3–5 s), fixation cross (5-6 s), and a portion of the remaining duration of solution receipt/consummatory phase (6–7.5 s). Note that increased 7–12 Hz power for water solution is specific to non-dorsolateral contacts during the post-cue fixation period.

We next conducted a set of control analyses to further examine the anatomical specificity of the low-frequency power cluster to the dlHPC region. We found that neither non-dorsolateral hippocampal contacts (Fig. 1d and Extended Data Fig. 2) nor visual areas (occipital, middle temporal; Extended Data Fig. 3) exhibit condition specificity in the low-frequency power cluster. Rather, recruited condition-specific power in these regions varied in the spectrotemporal dynamics. The absence of the low-frequency power cluster in visual areas further supports the notion that this cluster is not supporting mere generic visual processing. In the non-dlHPC, condition specificity was in a different peak frequency range (around 7–12 Hz) and time interval (post-cue, during the fixation cross), and power in this range was higher in the taste-neutral condition (Extended Data Figs. 4 and 5). It is possible that the immediate response observed in the dlHPC reflects the learned cue-rewarding taste association, whereas the late non-dlHPC response reflects anticipation of upcoming solution delivery or reflects neural dynamics underlying preparation for consumption.

**Extended Data Fig. 6:**
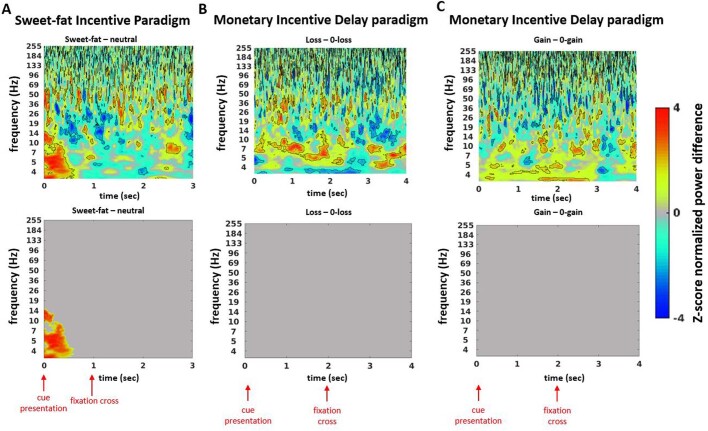
Cluster-based permutation testing during a period of anticipation of reward variables from two tasks with different contrasts in the dlHPC. (A) z-score normalized difference spectrograms between the sweet-fat minus the taste-neutral items in the sweet-fat incentive paradigm. Note, this is a repeat of Fig. 1c included here as a reference. (B-C) z-score normalized difference spectrograms in the Monetary Incentive Delay task (MID; see Fig. S1B), which provided two different contrasts: anticipation of monetary loss minus anticipation of 0-loss (B) and anticipation of monetary gain minus anticipation of 0-gain (C). Normalization for spectral power for the MID task was performed as described for the sweet-fat incentive paradigm. Note that the low frequency power cluster is specific to the sweet-fat cue and is not elicited by other visual cues associated with reward anticipation in the non-feeding domain (loss vs. 0-loss contrast and gain vs. 0-gain contrast).

**Extended Data Fig. 7:**
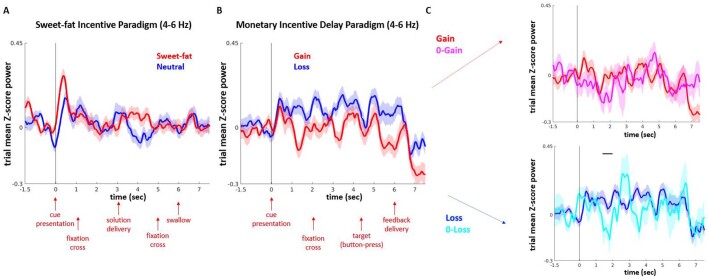
Power of 4–6 Hz during reward anticipation and receipt periods in two different tasks with different contrasts in the dlHPC hippocampal area. (A) 4–6 Hz power time traces for sweet-fat and taste-neutral items in the sweet-fat incentive paradigm. Note, this is a repeat of Extended Data Fig. 2, left included here as a reference. (B) 4–6 Hz power time traces for monetary-gain and monetary-loss trials in the MID paradigm. (C) 4–6 Hz power time traces for the gain and loss cues contrasted with their corresponding 0-gain (magenta) and 0-loss cues (cyan), respectively. The time interval displayed for the MID task includes the pre-cue period (−1.5 to 0 s), cue presentation (0–2 s), fixation cross (2–4 s), button-press target (~0.350 s within a 4–6 s interval), and a portion of feedback delivery (6–7.5 s). Note that increased 4–6 Hz power is specific to the sweet-fat vs. taste-neutral contrast.

**Extended Data Fig. 8:**
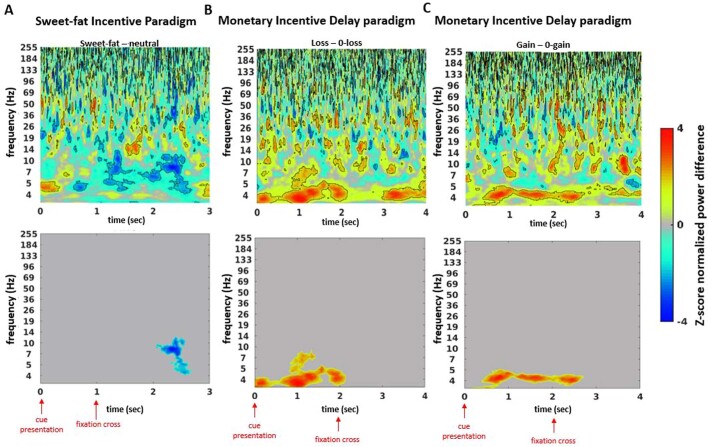
Cluster-based permutation testing during a period of anticipation of reward variables from two tasks with different contrasts in the non-dorsolateral hippocampal area. (A) z-score normalized difference spectrograms between the sweet-fat minus the taste-neutral items in the sweet-fat incentive paradigm. Note, this is a repeat of Extended Data Fig. 4a included here as a reference. (B-C) z-score normalized difference spectrograms in the Monetary Incentive Delay task (MID; see Fig. S1B), which provided two different contrasts: anticipation of monetary loss minus anticipation of 0-loss (B) and anticipation of monetary gain minus anticipation of 0-gain (C). Note that the two tasks recruit different spectral profiles in the non-dlHPC, which also differ from the task induced spectral profiles in the dlHPC (a double dissociation).

We next tested for the specificity of the low-frequency power cluster to food-related reward anticipation. Anticipation of a reward in a different context did not elicit the condition-specific dlHPC low-frequency power cluster. More specifically, visual cues in a different task—the monetary incentive delay task paradigm^17^ (Supplementary Fig. 1b), cueing receipt of monetary gain or loss (monetary reward anticipation and monetary loss anticipation, respectively)—did not elicit increased power in the low-frequency cluster in the dlHPC when compared with zero receipt (gain versus no gain and loss versus no loss) (Extended Data Figs. 6 and 7). Importantly, using these two task paradigms and the two hippocampal subdivisions (dlHPC and non-dlHPC), we found a double dissociation whereby two task paradigms make different processing demands on two dissociable subnetworks within the human hippocampus. Specifically, contrary to non-dlHPC processing of a delayed increase in lower-frequency power for taste-neutral items in the sweet-fat incentive task paradigm, robust and early increases in low-frequency power for both anticipation of gain and of loss of monetary reward were observed (Extended Data Fig. 8). Together, these results speak to the anatomical specificity of the dlHPC as an orexigenic subnetwork node and to the specificity of the low-frequency power cluster in this region to food-related appetitive processing.

## Evoked potentials between the LH and dlHPC

**Fig. 2:**
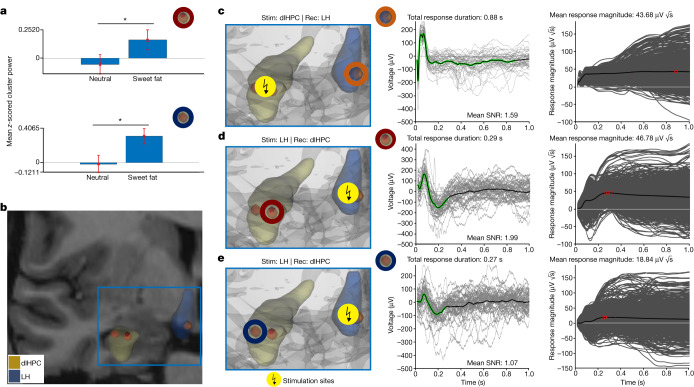
Dissecting the human LH–dlHPC appetitive processing circuit using single-pulse electrical stimulation. **a**, Increased mean *z*-score low-frequency cluster power in the dlHPC (two channels; top and bottom; outlined by red and dark blue circles) during anticipation of sweet-fat compared with taste-neutral items from a participant with electrodes implanted in both the dlHPC and LH area (*P* = 0.037 (top) and *P* = 0.009 (bottom), unpaired one-sided permutation testing, 1,000 permutations). Data are mean ± s.e.m. across trials in each channel (*n* = 33 trials per condition (top), and *n* = 33 and *n* = 30 trials for the taste-neutral and sweet-fat conditions, respectively (bottom)). **b**, The anatomical location of the dlHPC (yellow volume) and LH-area (blue volume) electrodes used in the trials of single-pulse electrical stimulation. We parameterized single trials and quantified response durations and magnitudes between the LH and dlHPC. **c**, Electrical stimulation (stim) was delivered through the electrode pair (the same electrodes as in **a**) in the dlHPC and elicited evoked potentials in the recording (rec) LH electrode outlined in orange. The extracted shapes of the evoked potentials (middle; black line with green highlighting) revealed initially sharp responses characterized by a mean magnitude of 43.68 μV *√*s. SNR, signal-to-noise ratio. **d**,**e**, The LH area also received electrical stimulation that elicited evoked potentials in the two recording dlHPC sweet-fat-responsive electrodes outlined in red and dark blue circles (the same electrodes as in **a**). **d**, The extracted shapes of the evoked potentials revealed responses with a mean response magnitude of 46.78 μV *√*s in the dlHPC electrode (outlined in red). **e**, The other dlHPC electrode, probably due to its location, had a lower mean response magnitude. *P < 0.05. 3D volumes were rendered using DSI Studio (v.2022; publicly available at https://dsi-studio.labsolver.org/). Source Data

Given that tractography enables only indirect assessment of interconnections and cannot assess the potential monosynaptic nature of interactions between two brain regions^18^, we performed trials of direct, single-pulse, electrical stimulation in a human participant with rare, if ever, recordings from both LH and sweet-fat-responsive dlHPC electrodes (Fig. 2a,b). Voltage deflections (or evoked potentials) are typically observed within 100 ms from the stimulation onset when recording from a region directly connected to the stimulation site^19,20^. We measured the evoked potentials (1) recorded in the LH after stimulation of a pair of sweet-fat-responsive electrodes in the dlHPC (Fig. 2c (left)) and (2) recorded in each of the two sweet-fat-responsive electrodes in the dlHPC after stimulation of the pair of electrodes that included the LH electrode (Fig. 2d,e (left)). The stimulation parameters were identical for all of the stimulation trials (bipolar, biphasic positive, 0.5 Hz, 6 mA, pulse width of 200 μs, 49 trials, 120 s total). We first identified that there was a significant reproducible response shape for each of the stimulation-recording iterations, and then parameterized single trials by the weight of the discovered shape and the residual noise (Fig. 2c–e) to calculate the duration of the significant responses and the mean response magnitudes—a metric that is not biased against longer-lasting responses (in contrast to methods using root-mean squared)^21^. After stimulation pulses to the sweet-fat-responsive dlHPC electrodes, we observed evoked potentials characterized by a fast, sharp, negative voltage deflection (~25 ms) and a slow return to the baseline, with a total duration of 0.88 s recorded in the single electrode within the LH area (Fig. 2c; see Supplementary Fig. 2 for raw, common average and bipolar rereferenced from single-trial signals). Similar stimulation pulses encompassing the LH area electrode also elicited evoked potentials characterized by an early positive deflection followed by a negative deflection and a return to the baseline, with a total duration of 0.27–0.29 s recorded in the two electrodes in the dlHPC (Fig. 2d,e; see Supplementary Fig. 3 for raw, common average and bipolar rereferenced from single-trial signals).

The extracted shapes revealed significant responses in both experimental directions (that is, recording in the LH with dlHPC stimulation and vice versa). Mean response magnitudes were similar between the LH electrode and one of the dlHPC electrodes (43.68 and 46.78 μV *√*s, respectively). Although we also recorded a significant response in the other dlHPC electrode, it had a lower magnitude (18.84 μV *√*s); however, the recordings from this electrode may have been affected by its location at the lateral dlHPC border, adjacent to the anterior horn of the lateral ventricle (Fig. 2b). This may also account, at least in part, for this electrode’s recordings having a lower mean signal-to-noise ratio (1.07) compared with the recordings from the LH electrode (1.59) and the first dlHPC electrode (1.99). These fast evoked potentials recorded in both regions after stimulation of the other are indicative of the presence of direct circuit-interactions between them, which may be bidirectional.

## MCH^+^ projections to dlHPC

**Fig. 3:**
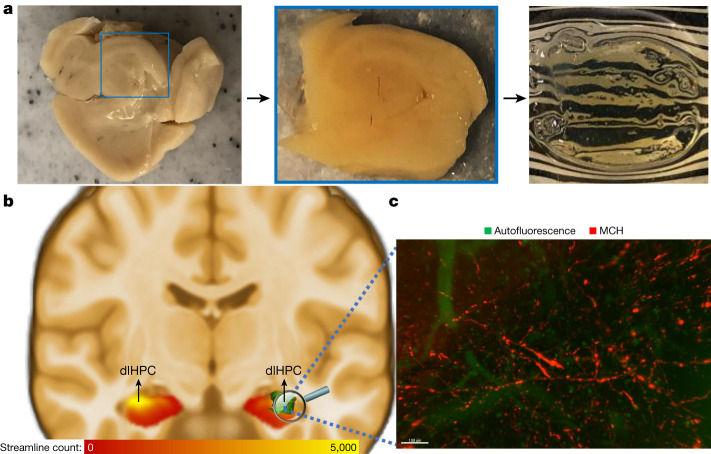
Dissecting the human LH–dlHPC appetitive processing circuit using 3D histology. **a**, Display of a post-mortem human sample (left) of the hippocampus and dlHPC section (middle) that was selected for the iDISCO brain-clearing (right) procedure. **b**, The iDISCO-cleared section (green) was overlaid to the group average dlHPC (yellow), defined on the basis of its higher number of LH streamlines. **c**, Staining for MCH^+^ and Alexa Fluor 647 is shown in red and autofluorescence in green within the dlHPC hotspot (high streamline probability with the LH area). The image was acquired using light-sheet microscopy (UltraMicroscope II). Scale bar, 100 μm. 3D visualization is shown in  Supplementary Video 1. This 3D histology experiment could not be repeated independently because only a single sample of the human dlHPC was available for the 3D histology experiments at our institution. We therefore approached these data as a unique opportunity for a proof of principle only, testing the feasibility of directly visualizing MCH^+^ LH projections with 3D histology (of which testing was lacking in humans).

Given that MCH is an orexigenic neuropeptide produced in the LH area with a well-described role in appetitive processing^5,10,11^, we next tested for MCH^+^ projections in the dlHPC subregion. To do so, we leveraged another rare opportunity afforded by a post-mortem sample of human tissue for the immunolabelling-enabled 3D imaging of solvent cleared organs (iDISCO) procedure (Fig. 3a (left)). This technique enabled 3D immunostaining and visualization of axonal projections carrying specific peptides within tissue cuboids, whereas conventional techniques would be limited in visualizing axons intersecting histological slices^22^.

**Extended Data Fig. 9:**
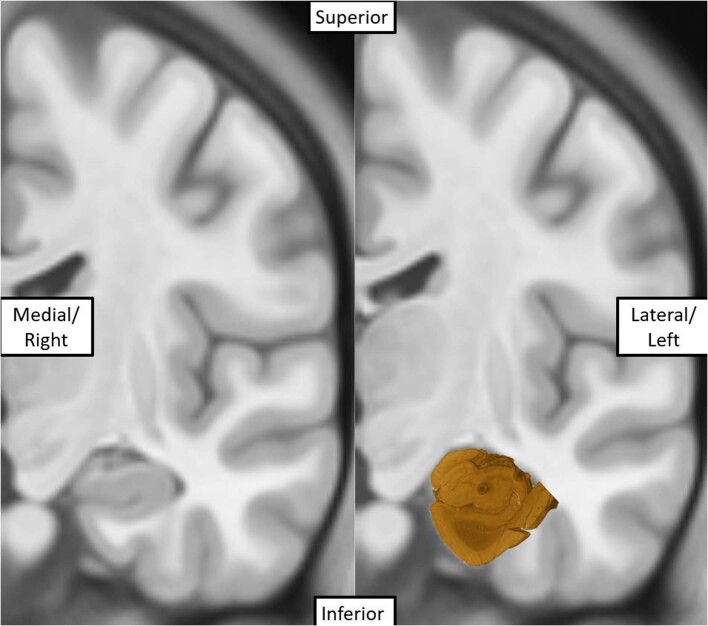
Manual localization of the ***post-mortem*** hippocampal sample in a coronal slice of the MNI 09c brain template.

First, we manually identified the location of our sample in a corresponding coronal slice in the high-resolution MNI 09c template brain (Extended Data Fig. 9). Second, we extracted a representative dorsolateral section that encompassed the dlHPC subregion in the template brain (Fig. 3a (middle)). This section was then processed according to the iDISCO brain-clearing procedure (Fig. 3a (right)), with staining for MCH (and Alexa Fluor 647). Third, the cleared and stained section was again manually overlaid to the corresponding coronal slice of the high-resolution MNI 09c template brain with the additional overlay of the tractography-identified dlHPC subregion (Fig. 3b). We found that the dlHPC section contained MCH^+^ orexigenic projections visualized using the brain-clearing 3D histology (Fig. 3c and Supplementary Video 1).

## LH–dlHPC is implicated in obesity

**Fig. 4:**
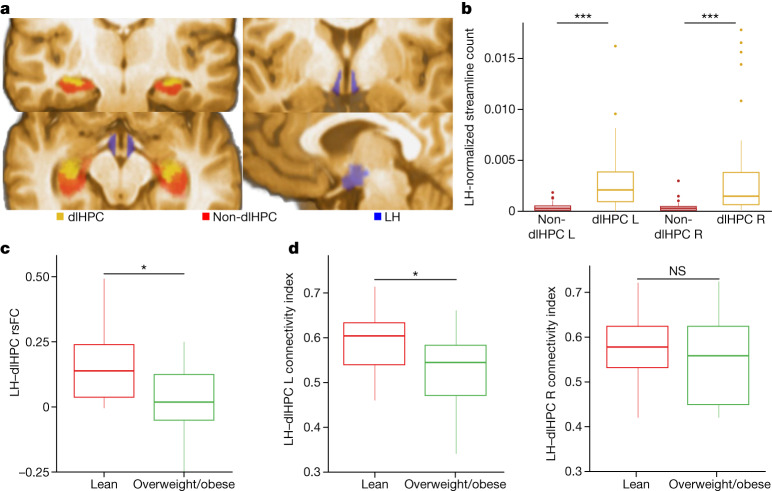
The dlHPC–LH circuit is associated with the obese state involving dysregulated eating behaviour in humans. **a**, Regions of interest co-registered to native space of an exemplary individual in the binge-eating cohort: dlHPC (yellow), non-dlHPC (red) and LH (blue; adapted from the CIT168 Subcortical In Vivo Probabilistic Atlas). **b**, Analysis of the relevance of hippocampal subregions in the binge-eating cohort. Significantly higher normalized streamline counts were observed between the LH and left (L) dlHPC (*t* = −4.585, *P* = 0.00006, two-sided *t*-test) and right (R) dlHPC (*t* = −3.609, *P* = 0.00097, two-sided *t*-test) compared with the non-dlHPC in the overall cohort. *n* = 34 participants, with 2 subregions analysed in each hemisphere. For the remaining analyses, the overall cohort was divided into two groups: lean (*n* = 17) and overweight/obese (*n* = 17). **c**, rsFC between the dlHPC and LH was decreased in the overweight/obese group compared with the lean group (*t* = 2.51, *P* = 0.018, two-sided *t*-test). **d**, Structural CI between the left dlHPC and LH was significantly decreased (*t* = 2.13, *P* = 0.042, two-sided *t*-test) in the overweight/obese group compared with the lean group. No significant differences (*t* = 1.07, *P* = 0.295, two-sided *t*-test) in the structural CI between the right dlHPC and LH were found (see Supplementary Fig. 4 for individual participant datapoints for **b**–**d**). NS, not significant. *P < 0.05; ***P < 0.001. For the box plots in **b** and **d**, the centre line shows the median, the box limits show the 25th to 75th percentiles and the whiskers show the minimum to maximum values. Source Data

Imaging data from a cohort of human individuals who are prone to binge eating (*n* = 34, female) were subdivided into overweight/obese (body mass index (BMI) ≥ 25 m^2^ kg^−1^; *n* = 17) and lean (BMI < 25 m^2^ kg^−1^, *n* = 17) groups. We confirmed in this cohort that the dlHPC contained the LH–dlHPC node, previously defined by LH streamlines, by co-registering our normative hippocampal subregions of interest and the atlas-based LH to images acquired from these human participants (Fig. 4a). Similar to the normative cohort described above (Fig. 1a), we found significantly higher normalized counts of LH streamlines in the left (*t* = −4.585, *P* = 0.00006) and right (*t* = −3.609, *P* = 0.00097) dlHPC voxels of this cohort compared with the hippocampal voxels outside the dlHPC (that is, non-dlHPC hippocampal voxels) (Fig. 4b).

**Extended Data Fig. 10:**
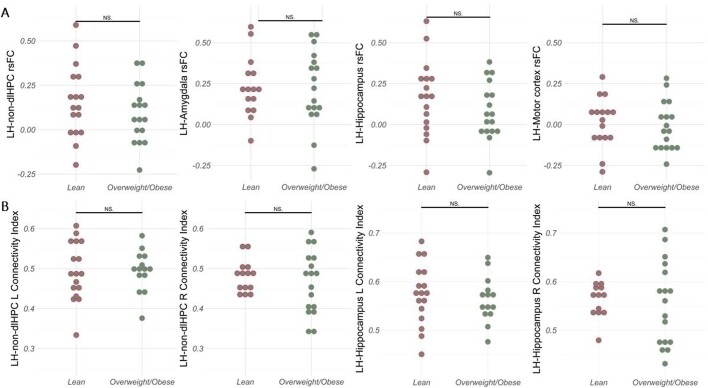
Connectivity of LH with control regions does not differ between overweight/obese and lean groups. (A) No significant differences in LH-non-dlHPC rsFC, LH-amygdala rsFC, LH-whole-Hippocampus or LH-motor cortex rsFC were found in overweight/obese compared to lean group (t-test, two-sided, unadjusted). (B) No significant differences in LH-non-dlHPC and LH-whole-Hippocampus structural CI were found in overweight/obese compared to lean group (t-test, two-sided, uncorrected). NS. = non-significant. Source Data

We next assessed whether structural and functional connectivity of the LH–dlHPC circuit differ between the overweight/obese and lean groups. We hypothesized that, specifically in a population with disordered appetitive processing, which can present as loss of eating control (that is, in individuals who are prone to binge eating), this circuit’s dysregulation may be directly related to excess weight^23,24^. This investigation was possible here only because we could ensure that the comparisons were performed between groups with similar demographics and patterns of disordered eating (Supplementary Table 3). We found that resting-state functional connectivity (rsFC) between the dlHPC and LH area was significantly decreased in overweight/obese compared with in lean participants (*t* = 2.51, *P* = 0.018; Fig. 4c). The probabilistic tractography-based structural connectivity index (CI) between the dlHPC and the LH area was also significantly decreased in the obese/overweight groups compared with the lean groups in the left (*t* = 2.13, *P* = 0.042) but not right (*t* = 1.07, *P* = 0.295) hemispheres (Fig. 4d). We confirmed that these connectivity findings were specific to the dlHPC subregion by performing a similar analysis between the non-dlHPC hippocampal voxels and the LH area. No differences in LH–non-dlHPC or LH–whole-hippocampus structural CI nor rsFC were observed between the overweight/obese and lean groups (Extended Data Fig. 10 and Supplementary Table 4 (also includes rsFC between LH and control regions)).

As we were ultimately interested in the overall multivariate pattern of these functional and structural circuit alterations, we fit a multivariate logistic regression model that included neuroimaging as well as behavioural variables (Methods; the variables are listed in Supplementary Table 3) to predict whether a participant belongs to the overweight/obese or lean group. Using backwards elimination, we identified LH–dlHPC rsFC (*β* = −9.886, *P* = 0.044) and LH–left dlHPC CI (*β* = −14.676, *P* = 0.037) as the only independent predictors of obesity, with a variance inflation factor (VIF) of 1.32 (VIF < 2.5 suggests negligible collinearity between variables). Such findings further implicate this circuit in obesity involving dysregulated eating behaviour.

## Discussion

As a higher-order processing centre involved in integrating external and internal stimuli, the hippocampus is uniquely positioned as an important node for orexigenic appetitive processing^1,2,25^. Here we characterized the orexigenic subnetwork within the human hippocampus. Structurally, LH streamlines converge in the dorsolateral aspect of the hippocampus (that is, the dlHPC); interconnections between the LH and dlHPC were further validated by single-pulse stimulation of the dlHPC resulting in sharp and fast voltage deflections in the LH area. This hippocampal subregion contains MCH^+^ projections that are presumably derived from the LH soma^5^. Functionally, the dlHPC exhibits specific field potential responses during anticipation of a high-caloric, sweet-fat solution. Finally, the LH–dlHPC circuit is perturbed in patients with obesity involving dysregulated eating patterns.

The interrogation of a neural circuit underlying appetitive processing in living humans poses unique challenges, and has mostly relied on functional MRI (fMRI) and non-invasive electrophysiology^26,27^. However, for over two decades, in vivo structural investigations of human brain circuits have been made possible by diffusion-MRI-based tractography^28^. The key limitation of tractography is that it may be prone to false positives and negatives, and it may also not allow distinction between afferent and efferent projections^29^. Nonetheless, tractography findings can be supported by direct interrogation of circuits with (1) stimulation-induced evoked potentials^30^ and (2) post-mortem brain clearing 3D histology^18^. Here we used high-resolution diffusion MRI to define the hippocampal subregion in which LH streamlines are more densely populated (that is, the dlHPC). Thereafter, we also applied the two aforementioned modalities uniquely in parallel to further probe and characterize LH interconnections within the dlHPC. Notably, the hereby defined human dlHPC subregion includes both posterior and anterior aspects of the human hippocampus; we therefore hypothesize that it is not analogous to the classical rodent subdivision of the dorsal and ventral hippocampus^31^.

Stimulation of either the dlHPC or the LH caused reproducible fast and sharp voltage deflections in the other region, indicating the presence of direct connections between them^19^. As our circuit hypotheses were driven by a wealth of previous literature in animal studies about a predefined interaction between the LH and hippocampus, we used a hypothesis-driven, preselected paradigm to study the dynamics between the human LH and dlHPC^20^. We used a technique that enables quantifications of the magnitude and duration of evoked potentials without a pre-set assumption of their form and shape in areas in which relative tissue to electrode positions and type of axonal projections are not yet well described^21^. Similar response magnitudes were recorded in the LH after dlHPC stimulation and vice versa. Importantly, responses to electrical stimulation do not necessarily follow the directions of axonal projections, as classically seen in the antidromic evoked potential stimulation recorded in prefrontal cortex after stimulation of the subthalamic nucleus, which reflects the hyperdirect pathway from the prefrontal cortex to the subthalamic nucleus^32,33^. Response shapes differed, including their latency and total duration, depending on which node received stimulation or recordings, potentially accounting for the inclusion of later changes in local field potentials recorded in the LH, as well as cytoarchitectonic differences between the two regions and the position of the electrode with regard to the tissue^20^.

In a post-mortem hippocampal specimen, we then used iDISCO 3D histology with immunostaining for MCH—a neuropeptide that is involved in feeding and primarily synthesized in the LH and its immediate adjacencies^5,10,11^—to further assess the presence of and characterize orexigenic LH projections within the dlHPC subregion. Given that we had only a single sample of the human dlHPC available for the 3D histology experiments, preventing the use of multiple stains in the same sample, we approached these data as a unique opportunity for a proof of principle, testing the feasibility of directly visualizing MCH^+^ LH projections with 3D histology (testing of which was lacking in humans). Our results provided additional evidence for the existence of such appetitive projections in the human dlHPC. Before 3D histology and immunolabelling, we were able to test the performance of the anti-MCH antibody, as described in the original iDISCO paper^22^, with and without the secondary antibodies. While rigorous quantifications of the visualized MCH^+^ staining would require multiple samples with comparable dimensions (and antibody permeability), the presented approach has the potential to inform at least one directionality of the identified LH–dlHPC subnetwork. These inferences were possible here due to previous understanding of the LH origin of MCH^+^ projections that enabled one assessment of directionality, which is rarely possible in human circuit-based investigations. These histologically defined LH projections within the dlHPC subregion shed light on the direction of the previously described evoked potentials, with the responses recorded in the LH area (after dlHPC stimulation) probably representing, at least in part, antidromic effects of stimulating these projections, as previously described in different subnetworks^32^. Importantly, the projections from the LH to the dlHPC uncovered by the 3D histology should not be regarded as the only direction of connections between these two regions. In fact, the opposite pathway has also been described in rodents, with a robust projection from the hippocampus to the LH also involved in the control of feeding^6,34^.

Comparable to our work, the sweet-fat paradigm used here has been applied to fMRI studies that showed increased hippocampal activation in response to sweet-fat stimuli compared with taste-neutral stimuli^35^. Moreover, hippocampal activation in response to food stimuli has been reported to be decreased after intranasal insulin administration^36^. Although these studies place the human hippocampus at the intersection of energy homeostasis and appetitive processing, fMRI and non-invasive electrophysiology lack the temporal resolution and spatial resolution, respectively, for uncovering differential hippocampal subregion involvement. Moreover, reports on hippocampal connectivity underlying dysregulated eating and obesity are lacking, and conspicuously absent are studies examining hypothalamic inputs in humans. Individuals undergoing brain mapping with intracranial electrophysiology provide a unique opportunity to overcome these limitations in the interrogation of specific regions of interest during controlled assays such as a food-incentive paradigm^12^.

Intracranial electrophysiology of this dlHPC subregion revealed a condition-specific power increase in the low-frequency cluster. The power in this range did not generalize to other brain areas nor other visual reward cues in the non-feeding domain. This frequency range overlaps with theta ranges, prominent rhythms in both the rodent and human hippocampus ascribed to mnemonic processes, including memory encoding and retrieval^37,38^. Our finding of increased dlHPC power as a function of trial number for the sweet-fat condition suggests that power in this range may support the encoding of the cue’s appetitive value. Lower frequencies, including theta, have also been observed in neocortical areas and have been linked to both mnemonic and cognitive control processes^39^. The ubiquitous presence of this rhythm across areas and behavioural contexts led to a hypothesis of its more general role, such as mediating information transfer between the recruited regions and at temporal scales associated with a given behavioural context^40^. The hippocampus is a higher-order node and, in the sweet-fat paradigm, is probably integrating multimodal information coming from the LH and neocortical areas; this low-frequency profile may mediate information transfer between the dlHPC and LH to facilitate combining cue information with appetitive input from the LH. Owing to the limited availability of patients with intracranial recordings for research tasks and limited time for such experiments, we were not able to directly assess the potential effect of satiety levels on these recordings beyond examining the task-period-related changes in energy state. Our report of a low-frequency power increase in this time window is consistent with power increase in the same frequency range in mice exposed to olfactory sweet-fat cues, reflecting a degree of generality of this signal to appetitive food anticipation irrespective of the sensory modality of the cue^41^.

Previous studies have also implicated the human hippocampus and hypothalamus in dysregulated eating and obesity. A recent systematic review of neural correlates of dysregulated eating associated with obesity risk in youth identified that eating in the absence of hunger was associated with hippocampal activity^42^. Hippocampal activation in response to food stimuli increased in children with obesity and dysregulated eating (for example, eating in dissonance with homeostatic requirements)^43^. Another study found that participants exposed to appealing food with a prior directive to suppress the desire for food significantly decreased activation in the hippocampus, among other regions involved in emotional regulation, conditioning and motivation^44^. Other studies reported a decrease in hippocampal activation in response to food images that predicts post-task levels of chocolate consumption, and abnormal hippocampal activation during reward processing in individuals with dysregulated eating behaviours^45,46^. In addition to the functional and structural neuroimaging studies, hippocampal concentrations of metabolites (such as creatine and phosphocreatine) have also been reported to be increased in individuals who are overweight or obese, potentially indicating BMI-related alterations in inflammatory cytokines and adipokines within the hippocampus^47^. With regard to the hypothalamus, previous research established an association between MCH overexpression and obesity in animal models^10^, and neuroimaging work reported increased hypothalamic activation during a task requiring inhibitory control in individuals with dysregulated eating^48^. Only one study has reported abnormal rsFC between the LH and multiple brain regions in adolescents with excessive weight^49^; while the hippocampus was identified as functionally connected to the LH, this subnetwork’s involvement in obesity was not reported. However, our finding of this link is probably due to the identification of the dlHPC as the subregion of interest, as findings of decreased LH–hippocampal connectivity in the obese state were not observed when hippocampal voxels outside the dlHPC were included.

The present investigation provides evidence supporting decreased rsFC and structural connectivity between LH and dlHPC in female individuals who are obese or overweight. These findings emerged only after the definition of the dlHPC as the subregion of interest, as our two cohorts showed no differences in connectivity between the non-dlHPC (or whole hippocampal) voxels and the LH. Moreover, the finding that functional and structural connectivity measures were significant predictors of overweight/obese versus lean group assignments supports the notion that the LH–dlHPC appetitive processing node is indeed altered in the obese state. Putting our findings in the context of the reports discussed above, structural and functional abnormalities involving the MCH^+^ LH–dlHPC node uncovered here may predispose individuals struggling with dysregulated eating behaviour to obesity. Our analyses with control regions indicated some specificity of the decreased LH–dlHPC connectivity in overweight/obese group, as opposed to reflecting a general effect of adiposity. Notwithstanding, our study findings of decreased LH–dlHPC connectivity in individuals who are overweight/obese in the context of binge eating should be interpreted with caution. The generalizability of these findings outside the context of disordered appetitive processing could not be assessed in the study cohort. It should also not be inferred from these results that this appetitive processing circuit is the only part of a large brain network associated with obesity. This study adds instead to the pre-existing animal literature with human findings implicating the LH–dlHPC circuit in appetitive processing.

There are a few noteworthy considerations regarding this study. First, we did not include male individuals or people struggling with other forms of dysregulated eating in the connectivity analyses. We chose to use a homogeneous female-only binge-eating cohort to ensure that the findings would not be skewed by sex or behavioural differences. However, our electrophysiological analysis is not sex specific. Second, directionality cannot be inferred from our cortico-subcortical evoked potential experiment, as the observed LH signal can be explained by either retrograde activity along the LH axons, or anterograde activity along the dlHPC axons. Third, MCH^+^ projections may be coming from either the LH or its immediately adjacent structures, such as the zona incerta^5^. Fourth, we had only a single case with simultaneous LH and dlHPC intracranial electrodes for the experiment with cortico-subcortical evoked potentials and a single sample for the 3D histology; however, together, these complimentary methods offered us a unique opportunity to leverage and cross-validate these approaches to examine a specific human orexigenic subnetwork. Finally, note that intracranial electrophysiological data are obtained from patients with epilepsy. Epilepsy may alter LH–dlHPC dynamics; however, previous research shows that normal physiological responses are recorded in patients with epilepsy outside of epileptic electrophysiological events^50^. Our analysis pipeline follows previous efforts to detect these events and eliminates any trial with at least one event. As epilepsy pathophysiology and foci vary from individual to individual, our group analyses limit the degree to which varied pathological dynamics are preserved at the group level. This suggests that our findings are useful for inferences about dlHPC–LH dynamics, but future validation regarding the generalizability of these findings to a non-epilepsy cohort is necessary.

Collectively, the convergence of modalities has elucidated a circuit that is perturbed in a disease-relevant state, furthering our understanding of how specific node interactions within the human brain are involved in obesity and related eating disorders.

## Methods

### MRI data and preprocessing

MRI acquisition parameters are summarized in Supplementary Table 2. We included MRI data from two different cohorts: (1) a normative diffusion MRI dataset from 178 unrelated individuals from the HCP who underwent a ultrahigh-resolution acquisition on the Magnetom 7T MRI scanner (Siemens Medical Systems) was obtained from the publicly available S1200 WashU-Minn-Ox HCP dataset^51–53^; (2) functional resting-state, and diffusion MRI data from 37 female individuals prone to binge eating recruited by the Stanford Eating Disorders Program on a 3T MRI scanner (Discovery MR750, GE Healthcare). Imaging data were analysed using publicly available methods and custom scripts in Python v.3.6, as described below.

Resting-state fMRI scans from the binge-eating-prone cohort were preprocessed using fMRIPrep (v.1.2.3)^54^. In brief, the preprocessing of the functional image involved skull-stripping, co-registration to the T1 reference image, and head motion and susceptibility distortion corrections. After removal of non-steady state volumes and spatial smoothing with a 6 mm FWHM isotropic Gaussian kernel, ICA-AROMA was used to identify motion-related noise components in the BOLD signal^55^. Framewise displacement (FD) and root-mean squared variance over voxels of the temporal derivative of time courses (DVARS) were calculated^56,57^. Three participants were excluded due to excessive movement as measured by (1) mean FD > 0.2mm; (2) more than 20% of FD over 0.2 mm; or (3) any FD > 5 mm (ref. ^58^). Global signals were extracted within the cerebrospinal fluid, white matter, grey matter and whole-brain masks. XCP Engine v.1.0 was used to perform denoising of the preprocessed BOLD output from fMRIPrep, using the estimated confound parameters^58,59^. This included demeaning and removal of any linear or quadratic trends and temporal filtering using a first-order Butterworth bandpass filter (0.01–0.08 Hz). These preliminary preprocessing steps were then followed by confound regression of ICA-AROMA noise components, together with mean white matter, cerebrospinal fluid and global signal regressors. All regressors were band-pass filtered to retain the same frequency range as the data to avoid frequency-dependent mismatch^59^. Whereas preprocessing was performed on the diffusion MRI data from the binge-eating-prone cohort to prepare the images for probabilistic tractography using the FSL suite^60,61^, the normative HCP diffusion MRI data had already been preprocessed (with the minimal preprocessing pipeline). The diffusion-weighted images were corrected for motion and geometric distortions using the topup and eddy functions, similar to that applied in the HCP’s preprocessing pipeline. For each participant, diffusion and T1-weighted images were co-registered using boundary-based registration.

### Probabilistic tractography

Probabilistic tractography was used to evaluate the interconnections between the LH and hippocampus. The LH mask was defined on the standard T1 MNI152 09c template adapted from CIT168 Subcortical In Vivo Probabilistic Atlas^62^, whereas the hippocampus mask was defined using the Harvard–Oxford Brain Atlas. Co-registration was performed using Advanced Normalization Tools (ANTs, v.2.1.0), and consisted of two successive steps of linear and nonlinear registration between the individual’s brain and the MNI brain. In a third step, the MNI-defined regions of interest were registered to the individual’s space. FSL’s Bayesian estimation of diffusion parameters obtained using sampling techniques (BEDPOSTX) was used to conduct Monte Carlo sampling of probability distribution of diffusion parameters at each voxel, accounting for up to three crossing fibre directions within a voxel^63^. Fibre tracking was performed using FSL’s Probtrackx2, using distance correction and each hippocampal voxel as a seed and the LH as a target^64^. A total of 5,000 seed points was used to generate streamlines from each seed voxel, and only the streamlines that reached the target were retained for further analysis. The results of Probtrackx are summarized in a map of ‘streamline probability’ and ‘waytotal’, representing the probability for each seed voxel to reach the target and the total number of streamlines from a given seed that reached the target, respectively. The strength of the connections between seed and target was calculated as a tractography-CI, as defined in a previous study using the folllowing formula: log[waytotal]/log[5,000 × Vseed] (ref. ^65^). The waytotal resulting from the tractography was log-transformed and divided by the log-transformed product of the generated sample streamlines in each seed voxel (5,000) and the number of voxels in the respective seed mask (Vseed). The log-transformation increased the likelihood of reaching normality, which was tested using the Shapiro–Wilk test^66^.

### Hippocampal segmentation

Tractography was used to generate a probabilistic map based on each hippocampal voxel’s streamline probability to the LH for all 178 participants from the normative HCP dataset. Each participants’ streamline probability map to the LH was transformed to standard MNI 09c space so that they could be averaged and concatenated into a normative weighted average group map of streamline probability between the hippocampal area and the LH across the 178 HCP individuals. We performed this analysis to define the hippocampal subregions in the normative HCP data and then applied these subregions to the binge-eating-prone cohort. We then used *k*-means to segment group average hippocampus streamline probability maps. This hypothesis-free method uses successive iterations to assign each voxel to one of two clusters without the application of external spatial constraints. For the case of large intervoxel similarities in streamline count, the algorithm does not identify two distinct clusters. Resulting clusters represented normative hippocampal subregions based on its connectivity to the LH in standard MNI 09c space. Finally, we co-registered the normative clusters to the MRI images from our participants implanted with depth electrodes, as well as from the members of the binge-eating cohort.

### Sweet-fat incentive and monetary incentive delay paradigms

Consent to participate in this study was obtained according to the Declaration of Helsinki and approved by the institutional ethical committee. The inclusion criteria for this study were the presence of at least one hippocampal depth electrode. Participants (*n* = 9; Supplementary Table 1) underwent surgical implantation of depth electrodes for neurosurgical epilepsy monitoring. The location of electrode implantation was determined solely based on clinical needs and therefore varied across participants. All of the patients provided individual informed consent (including the publication of de-identified demographics and clinical data) as approved by the Stanford University Institutional Review Board (IRB-11354). Of the 9 participants, 8 participants also had electrodes in regions specifically included in control analyses: 8 participants with electrode in the middle temporal gyrus and 2 participants with electrodes in the occipital lobe. Data acquisition procedures were previously described^12^. In brief, neural activity was sampled at 1,024 Hz from AdTech electrodes while the participants engaged in two different tasks—the sweet-fat incentive and monetary incentive delay computer-based paradigms (Supplementary Fig. 1). The sweet-fat incentive paradigm, also known as the Milkshake task^67^, was originally an fMRI task that we previously adapted for intracranial electrographic recordings of cued anticipation and consumption of a sweet-fat and a taste-neutral solution^12,68^. Each trial in this paradigm began with a 2 s fixation cross presented on a computer screen—this period is referred to as the prestimulus period. This was followed by a 1 s presentation of an image of a glass of either water or of milkshake, which served as a cue for the solution to be subsequently delivered through a mouthpiece to the participant for consumption. Before the solution was delivered, a 2 s image of a fixation cross was viewed. The 1 s presentation of the solution to be delivered and this 2 s fixation cross period are referred to as an anticipatory period (3 s). After the anticipatory period is a 5 s receipt/consummatory period, consisting of a 3 s solution-delivery period followed by a 2 s consumption of solution period. Sweet-fat and taste-neutral trials were presented in a randomized order, with a total of 80 to 100 trials evenly split between the sweet-fat and taste-neutral conditions. After task completion, the participants were asked to rate on a Likert scale from 1 to 10 the quality of the sweet-fat solution (Likert scale, 1–10) and which solution (sweet-fat versus taste-neutral) they preferred. The second task, the monetary incentive delay task paradigm, was also originally an fMRI task^17^ that we adapted for intracranial electrographic recordings of cued anticipation and receipt of monetary reward. Each trial in this paradigm is a total duration of 10 s. Trials began with a 2 s fixation cross presented onto a computer screen (prestimulus period). This was followed by a 2 s presentation of an image cueing the outcome of a subsequent button press as either monetary gain (+US$5, +US$1), absence of monetary gain (+US$0, referred to as zero-gain), monetary loss (−US$5, −US$1), or absence of monetary loss (−US$0, referred to as zero-loss). The cue is then followed by a 2 s display of a fixation cross image. The target image is presented momentarily within a 2 s interval prompting the participant’s button press. After the button press, there is a 2 s feedback presentation indicating gain, loss or absence of gain or loss. Gain and loss trials were presented in a randomized order and evenly split among all of the trial conditions. The total number of trials varied from 60 to 100 trials based on time constraints.

### Electrode localization

Presurgical MRI scan was co-registered to post-surgical computed tomography scan for electrode visualization and localization as described previously^50^. Locations of depth electrodes within the medial temporal lobe were then examined by one rater with expertise in medial temporal lobe anatomy and neuroimaging (D.A.N.B.). Electrodes in direct contact with the hippocampal area were selected for further evaluation. We next co-registered the normative hippocampal clusters (that is, dlHPC and non-dlHPC) that we had previously defined in the standard MNI09c template brain to each participant’s native space (Fig. 1e). All hippocampal electrodes (*n* = 54) were labelled according to whether they were in direct contact with dlHPC or not (that is, non-dlHPC). This localization was performed before the time–frequency analysis.

### Task data preprocessing and analyses

Electrophysiological data were downsampled to 1,000 Hz, notch filtered for 60 Hz and 2nd–3rd harmonics, and Laplacian rereferenced in FieldTrip as previously described^12,68^. Artifact timepoints were defined as voltage values greater or less than the mean signal of all 10 s trials concatenated, recorded from the same channel plus four multiples of its s.d. Any trial with at least one detected artifact timepoint was excluded. Time–frequency analysis was implemented using the wavelet toolbox in MATLAB. There are three input parameters: (1) minimum frequency, set to 3; (2) maximum frequency, set to 250; and (3) NumVoices, set to 32. The toolbox generates ‘scales’ on the basis of the desired frequency range (defined by minimum to maximum frequencies), which then get mapped into frequencies. The trial vector, scales vector and ‘morl’ are inputs to the cwtft function in MATLAB, which generates the wavelets and power extraction. Wavelets were first tested on ground-truth data with known spectral properties before use on experimental data.

Trial instantaneous power values were normalized to power at the same frequency and channel during the 1 s prestimulus period across all trials in the same condition (condition-specific prestimulus normalization). The prestimulus duration of any trial with at least 1 detected artifact timepoint was excluded from the normalizing distribution (see the previous section). Condition-specific prestimulus normalization was used to account for possible differences in baseline power before stimulus presentation. The results were reproduced using an alternative normalization method whereby power values were normalized relative to the distribution of power in the same frequency and channel, during the entire recording.

Spectral analyses were primarily focused on the anticipation period (1 s cue, and 2 s post-cue fixation for the sweet-fat incentive paradigm or 2 s cue and 2 s post-cue fixation for the monetary incentive delay paradigm). Statistical differences in time–frequency power between the conditions were calculated using cluster-based permutation testing^69^. In brief, this involved calculating a *t*-statistic in each time–frequency voxel, between the two conditions (sweet fat versus taste neutral), thereby generating the observed *t*-map. The distribution for each voxel was generated by pooling the time–frequency maps from all channels and individuals (trials were averaged to generate a single map per channel). The observed *t*-map was then compared to a null distribution (shuffled condition labels) of *t*-maps generated over 1,000 paired permutations. A *P* value for each voxel was obtained by comparing the observed to the null *t*-value at the same time–frequency voxel, thereby generating a *P-*map. Clusters of contiguous voxels with a *P* < .05 were identified and compared to the null-distribution cluster size. Observed clusters with sizes larger than the 95th percentile of those from the null distribution were considered to be significant after correction for multiple comparisons.

### Evoked potentials

As previously described, we performed single-pulse stimulations at rest using an intracranial electrical waveform generator and switchbox^30,70^ (MS-120BK-EEG and PE-210AK, Nihon Kohden). Electrical stimulation was delivered through adjacent pairs of electrodes in biphasic pulses (6 mA; 200 μs per phase, 49 trials) at a frequency of 0.5 Hz for a total of 120 s. We measured electrical potentials in response to stimulation with a video EEG monitoring system using a sampling rate of 2,000 Hz (version WEE-1200, Nihon Kohden). We analysed the single-pulse stimulation data using custom scripts in MATLAB v.2020b. We first applied a high-pass butterworth filter (1 Hz) to exclude slow varying effects and segmented evoked responses time series from recording channels were into 2,500 ms epochs time-locked to stimulus onset (500 ms prestimulus and 2,000 ms post-stimulus). We then rereferenced the data to the common average signal, excluding stimulated channels, channels with artifacts and channels with large, evoked responses, as previously described^20^. Finally, to exclude potential effects of prestimulus signal fluctuations, we applied a baseline correction by subtracting the average signal between 200 ms and 20 ms before the stimulus onset. To ensure that these preprocessing steps did not introduce a bias, we also provided line traces of bipolar rereferenced and the single-trial raw signal from the recording electrodes (Supplementary Figs. 2 and 3). To quantify the observed evoked potentials, we used the publicly available canonical response parameterization method and calculated the duration of the significant responses and mean response magnitudes, which is a metric that is not biased against longer-lasting responses (unlike methods using root-mean squared)^21,71^. This method automatically processes a set of responses recorded after repeated trials of stimulation and extracts a canonical structure in the response (if one exists), without a pre-set assumption of the response shape, to examining structural similarity between trials to (1) identify whether there is a significant reproducible response shape (and over what time interval); (2) characterize what this shape is; and (3) parameterize single trials by the weight of the discovered shape and the residual after the discovered shape has been regressed out. The output of this method consists of the magnitude, duration and significance of response to stimulation between pairs of brain sites.

### Human hippocampal sample

In accordance with the local Stanford University Institutional Review Board, a post-mortem sample of the left hippocampal area (Fig. 3a) was obtained from a whole brain with no known pathologies that had been extracted 24 h after death and placed in 10% formalin for 1 day. The sample was perfused and stored in PFA 4%. For the brain-clearing procedure, we extracted a representative dorsolateral hippocampal section and transferred the sample to 1 M phosphate-buffered saline (PBS). The sample was stored at 4 °C until the iDISCO protocol was performed.

### Antibody validation protocol

A validation protocol was performed to be sure that the anti-MCH (Phoenix Pharmaceuticals, H070-47, 01629-10) was compatible with the reagents used in the iDISCO protocol^22^. Slices of human hippocampal tissue (outside the dlHPC cuboid) were obtained on a Vibratome at 60 µM in 1 M PBS solution. The free-floating sections were permeabilized for 3 h with methanol at room temperature and after were rinsed twice with PBS for 20 min and then rinsed with PBS with 2% Triton X-100. The sections were then incubated with permeabilization solution (PBS with 0.2% Triton X-100) during 30 min and blocking solution (PBS with 0.2% Triton X-100, 10% DMSO and 6% donkey serum) for 1 h. The anti-MCH antibody was incubated 1:500 in PTwH (PBS with 0.2% Tween-20, 1% heparin (10 mg ml^−1^), 0.2% sodium azide) overnight at −4 °C. The samples were rinsed three times for 5 min and the secondary antibody (Alexa Fluor Plus 647 anti-rabbit; Thermo Fisher Scientific, A32795, TF271041) was incubated 1:250 in PTwH and 3% donkey serum and 0.2% sodium azide during 1 h at room temperature, covered from the light. Finally, the samples were rinsed in PTwH three times for 5 min and the slices were mounted with DAPI (Vectashiels-VECTOR). Images were acquired using confocal microscopy (data not shown).

### iDISCO brain-clearing 3D histology

After antibody validation, we selected a representative dorsolateral section for the iDISCO protocol to confirm our hippocampal area segmentation (Fig. 3a,b). The section was approximately 1.0 × 0.8 × 0.4 cm and was pretreated with methanol according to the iDISCO protocol^22^ using a modified immunostaining protocol. The sample was rinsed twice with PBS with 2% Triton X-100 for 1 h. We incubated the sample in permeabilization solution (PBS with 0.2% Triton X-100, for 30 min) and blocking solution (PBS with 0.2% Triton X-100, 10% DMSO and 6% donkey serum) for 1 h. The sample was incubated with 1:500 anti-MCH antibodies in PTwH (PBS, 0.2% Tween-20, 1% heparin 10 mg ml^−1^, 0.2% sodium azide) for 10 days, nutating at 37 °C. After 10 days, the samples were rinsed three times for 5 min and then rinsed again every few hours and left nutating at room temperature overnight. The next day, the sample was incubated in the secondary antibody, donkey anti-rabbit Alexa Fluor 647 Plus (Thermo Fisher Scientific, A32795), 1:250 in PTwH and 3% donkey serum and 0.2% sodium azide at 37 °C, nutating for 10 days, covered from the light. After secondary incubation, the sample was again rinsed in PTwH for 2 days, and the iDISCO clearing protocol was followed (https://idisco.info/idisco-protocol/update-history/).

### Histological assessment of lateral hypothalamic connections in the dlHPC

The iDISCO brain-clearing 3D histology results were used to confirm the hippocampal area segmentation. The anti-MCH antibody was used to identify orexigenic projections within our hippocampal sample. The sample was imaged using a light-sheet microscope (UltraMicroscope II, Miltenyi BioTec). We used the background subtraction tool in Imaris to remove the faint auto-fluorescence signal in non-stained tissue traditionally present in the red (647) channel of iDISCO-cleared samples. We assessed whether our sample from the dorsolateral hippocampal subregion, defined on the basis of the higher number of probabilistic tractography streamlines, contained projections expressing MCH, an orexigenic neuropeptide primarily produced in the LH area. We first manually identified the location of our whole sample in a corresponding coronal slice in the high-resolution MNI 09c template brain (Extended Data Fig. 9) and then extracted a representative dorsolateral section that overlapped with the dlHPC (Fig. 3a). After immunolabelling and clearing, the final MCH-stained sample was again manually overlaid to the corresponding coronal slice in the high-resolution MNI template brain and the tractography-based hippocampal probability map of LH area streamlines (Fig. 3b).

### Demographics, clinical and behavioural data of the binge-eating-prone cohorts

Participant consent was obtained according to the Declaration of Helsinki and approved by the Stanford University Institutional Review Board (IRB-35204). We analysed the available clinical and behavioural data from the 34 female individuals who were prone to binge eating (that is, all those included in imaging analyses), defined by at least one weekly episode of eating large amounts of food in short periods accompanied by the feeling of loss of control eating over the previous 6 months (binge-eating-prone cohort; mean age = 26 ± 5.6 years; BMI = 27.9 ± 8.5; binge frequency = 2.7 ± 1.4 episodes per week)^72^. The number of binge-eating episodes per week was assessed using the Eating Disorder Examination, a standardized diagnostic interview^73^. The Beck Depression Inventory (BDI) and the Beck Anxiety Inventory (BAI) were used to screen for depression and anxiety, respectively^74,75^. The Difficulties in Emotion Regulation Scale was used to assess impairment in emotion regulation^76^. The binge-eating cohort was divided into two subgroups: (1) lean (*n* = 17): BMI < 25 (referred to as the lean group); (2) overweight/obese (*n* = 17): BMI > 25 (referred to as obese/overweight group).

### rsFC analysis

rsRC analysis was performed on the binge-eating-prone cohort’s preprocessed resting-state fMRI data using DPABI/DPARSF v.4.3, which is based on Statistical Parametric Mapping (SPM, v.12, https://www.fil.ion.ucl.ac.uk/spm)^77^. A seed-based approach was performed to examine the rsFC in the 34 female individuals prone to binge eating included in imaging analyses by calculating the rsFC between the LH mask as defined above and each tractography-identified hippocampal subregion. Functional connectivity values were extracted for all participants and used in further correlational analyses.

### Statistical analyses

Statistical analyses were performed using the Rstudio v.1.2.5042 (Rstudio). Given the sensitivity of metrics derived from resting-state fMRI and diffusion MRI proneness to numerical distortions related to data acquisition or analytical pipeline, we used the Tukey method to remove outliers for each connectivity metric. After checking for normality, we then used the Student’s *t*-test to compare rsFC as well as tractography-CI between the hippocampal subregions and LH in the overweight/obese and lean groups. One outlier was identified and removed from the obese binge-eating group and two outliers were identified and removed from the lean group in both the rsFC and structural CI analyses in Fig. 4c and 4d (left), respectively. Moreover, we identified and removed one outlier from the lean group in the CI analysis in Fig. 4d (right). Mann–Whitney *U*-tests were used to compare the corrected number of streamlines between the LH and hippocampal subregions in the binge-eating-prone cohort. To assess potential effect of confounders in the identified connectivity differences between the obese and lean group, we fit a multivariate logistic regression model to predict whether a participant belongs to the overweight/obese or lean group, including the available demographic and behavioural variables in addition to the LH–dlHPC connectivity measurements. The comprehensive list of variables included: age, depression (BDI), anxiety (BAI), binge-eating frequency, restrained eating, emotional eating and externally driven eating scores (from DEBQ), LH–dlHPC–LH rsFC, LH–non-dlHPC rsFC, LH–dlHPC structural connectivity and LH–non-dlHPC structural connectivity. We then used backwards elimination to identify which combination of variables provided the highest predictive power with the lowest total number of explanatory variables to avoid over-fitting (Akaike information criterion). Finally, a VIF was calculated to assess potential correlations between explanatory variables, with a VIF < 2.5 suggesting negligible collinearity between variables^78^. *P* < 0.05 was considered to be statistically significant for all tests.

### Reporting summary

Further information on research design is available in the Nature Portfolio Reporting Summary linked to this article.

## Online content

Any methods, additional references, Nature Portfolio reporting summaries, source data, extended data, supplementary information, acknowledgements, peer review information; details of author contributions and competing interests; and statements of data and code availability are available at 10.1038/s41586-023-06459-w.

## Supplementary information

Supplementary Figures Supplementary Figs. 1–4.

Reporting Summary

Supplementary Tables Supplementary Tables 1–4.

Supplementary Video 1 dlHPC 3D histology sample localization and visualization. Animated 3D visualization of Fig. 3.

## Data Availability

The Human Connectome Project 7T S1200 WashU-Minn-Ox HCP dataset is publicly available online (http://db.humanconnectome.org/). More detailed anonymized data supporting any other findings of this study are available from the corresponding author on reasonable request as the institutions involved in this study may require Data Use Agreements, which we would be happy to facilitate for investigators who are interested in replicating (or expanding on) our findings. Source data are provided with this paper.

## Source data

Source Data Fig. 1
Source Data Fig. 2
Source Data Fig. 4
Source Data Extended Data Fig. 2
Source Data Extended Data Fig. 10

## Code availability

MATLAB (v.2020b), Python (v.3.6), FSL (v.6.0), Advanced Normalization Tools (v.2.1.0), fMRIPrep (v.1.2.3), DPABI/DPARSF (v.4.3) and SPM (v.12) were used for the electrophysiologic and neuroimaging analyses in this study. The code and method^21^ used to analyse the evoked potentials are also publicly available at GitHub (http://github.com/kaijmiller/crp_scripts). Imaris (v.8.4) was used to render histological images. Statistical analyses of the neuroimaging results were performed using Rstudio (v.1.2.5042). No new algorithms or pre-processing techniques were performed outside of the previously published uses of the toolboxes and software packages. The code used for analysing the data is protected under USPTO serial number: 63/170,404 and 63/220,432; international publication number: WO 2022/212891 A1 (international publication date: 6 October 2022) and can therefore be shared by the corresponding author only on reasonable request.
