# Supplementary material for: An orexigenic subnetwork within the human hippocampus
Source: Nature. 2023 Aug 30;621(7978):381–8. doi: 10.1038/s41586-023-06459-w (PMC10499606; doi:10.1038/s41586-023-06459-w)
Supplement: Supplementary file 1 — Supplementary Figs. 1–4. [file 41586_2023_6459_MOESM1_ESM.pdf]

nature portfolio

https://doi.org/10.1038/s41586-023-06459-w

Supplementary information

An orexigenic subnetwork within the human hippocampus

In the format provided by the authors and unedited

SI Figure 1 | Task design adapted for intracranial recordings. (A) Sweet-fat incentive paradigm. Each trial in this paradigm began with a 2-sec presentation of fixation cross (pre-stimulus period). This was followed by a 1-sec presentation of a drawing of a glass representative of either a taste-neutral or a sweet-fat solution, which served as a cue for the solution to be subsequently delivered. A 2-sec presentation of a fixation cross then preceded delivery of the solution. Following the anticipatory period is a 5-sec receipt/consummatory period, consisting of a 3-sec solution delivery period followed by a 2-sec consumption of solution period. (B) Monetary incentive delay task paradigm. Each trial in this paradigm began with a 2-sec fixation cross presented on a computer screen (pre-stimulus period). This was followed by a 1-sec presentation of an image of indicating the possibility of monetary gain (+$5, +$1), absence of monetary gain (+$0, referred to as 0-gain), monetary loss (-$5, -$1), or absence of monetary loss (-$0, referred to as 0-loss), which served as a cue for the outcome of a subsequent button press. After the button press, there is a 2-sec feedback presentation indicating gain, loss, or absence of gain/loss, followed by a 2-sec pre-stimulus period initiating the next trial.

SI Figure 2 | Time series from single trials of dlHPC-LH evoked potentials recorded in the LH in response to single-pulse stimulation of a sweet-fat-responsive, dlHPC electrode pair. Each color lines represent signal traces for a single stimulation trial (n=49) visualized after different pre-processing steps: (A) after applying high-pass filter, (B) common average re-referencing (CAR) with baseline-correction, (C) CAR without baseline correction, and (D) bipolar re-referencing without baseline correction.

SI Figure 3 | Time series from single trials of LH-dlHPC evoked potentials recorded in two sweet-fat-responsive in dlHPC. dlHPC channels (ch), identified as ch 127 and ch 128, in response to single-pulse stimulation of a pair of ipsilateral electrodes in LH area. Each color lines represent signal traces for a single stimulation trial (n=49) visualized after different steps of pre-processing: (A, B) after applying high-pass filter, (C, D) common average re-referencing (CAR) with baseline-correction, (E, F) CAR without baseline correction, and (G) bipolar re-referencing without baseline correction.

SI Figure 4 | Individual subject data points assessing the LH-dlHPC appetitive processing circuit with regards to the obese state in the context of dysregulated eating. A) Significantly higher normalized streamline counts observed between the LH and the left (t = -4.585; p < .00006; t-test, two-sided) and right (t = -3.609; p < .00097) dlHPC compared to the non-dlHPC in the overall binge-eating cohort. (B) rsFC between the dlHPC and LH was decreased in overweight/obese compared to the lean group (t = 2.51; p = .018; t-test, two-sided). (C) Structural CI between the left dlHPC and LH was significantly decreased (t = 2.13; p = .042; t-test, two-sided) in overweight/obese compared to lean group. No significant differences (t = 1.07; p = .295; t-test, two-sided) in structural CI between the right dlHPC and LH were found. NS. = non-significant. * = p < .05. *** = p < .001.

SI Figure 1

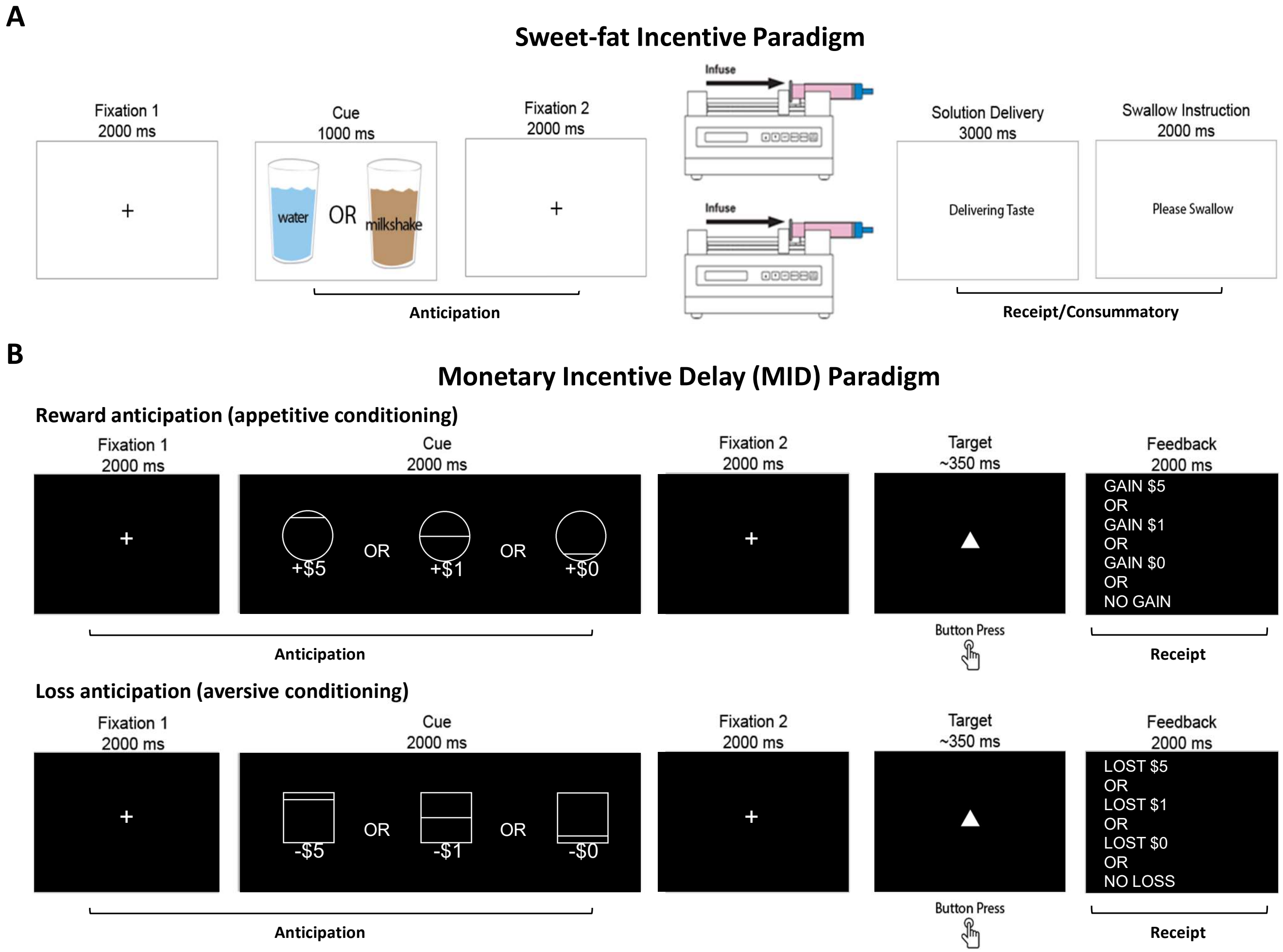
A
Sweet-fat Incentive Paradigm
Fixation 1
2000 ms
Cue
1000 ms
water
OR
milkshake
Fixation 2
2000 ms
Infuse
Infuse
Solution Delivery
3000 ms
Delivering Taste
Swallow Instruction
2000 ms
Please Swallow
Anticipation
Receipt/Consummatory
B
Monetary Incentive Delay (MID) Paradigm
Reward anticipation (appetitive conditioning)
Fixation 1
2000 ms
Cue
2000 ms
+$5
OR
+$1
OR
+$0
Fixation 2
2000 ms
Target
~350 ms
Button Press
Feedback
2000 ms
GAIN $5
OR
GAIN $1
OR
GAIN $0
OR
NO GAIN
Anticipation
Receipt
Loss anticipation (aversive conditioning)
Fixation 1
2000 ms
Cue
2000 ms
-$5
OR
-$1
OR
-$0
Fixation 2
2000 ms
Target
~350 ms
Button Press
Feedback
2000 ms
LOST $5
OR
LOST $1
OR
LOST $0
OR
NO LOSS
Anticipation
Receipt

SI Figure 2

Stimulation: dlHPC | Recording: LH

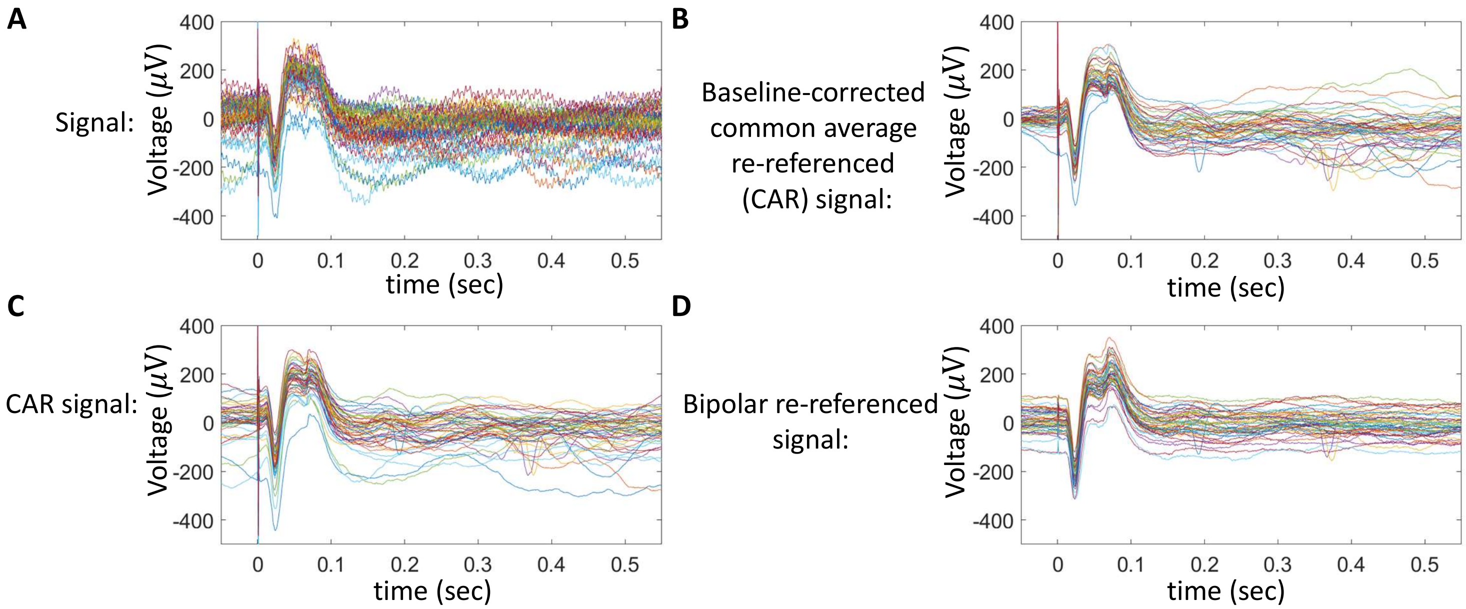

Stimulation: LH and adjacencies | Recording: dlHPC - channels (ch) 127 & 128 **SI Figure 3**

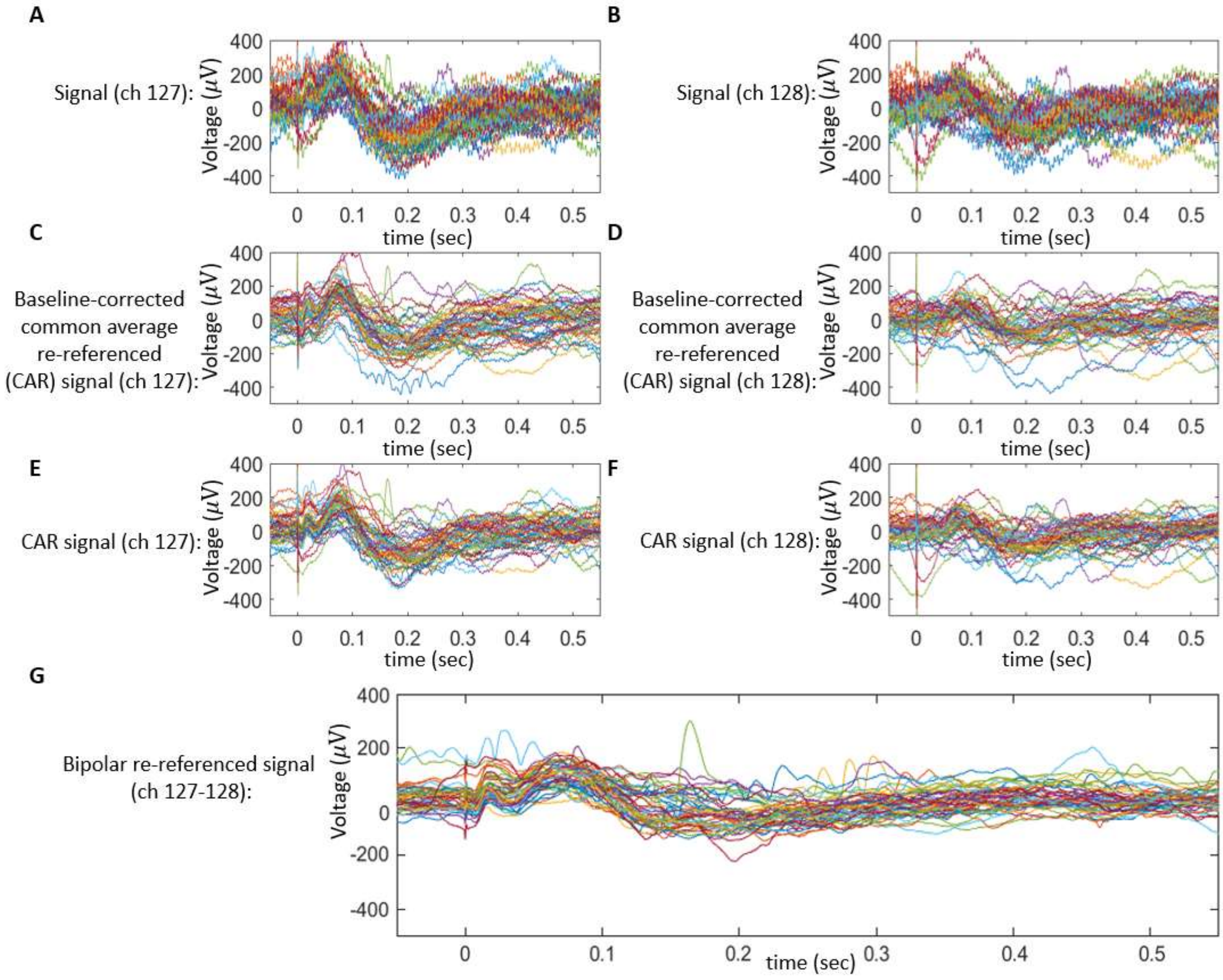

SI Figure 4

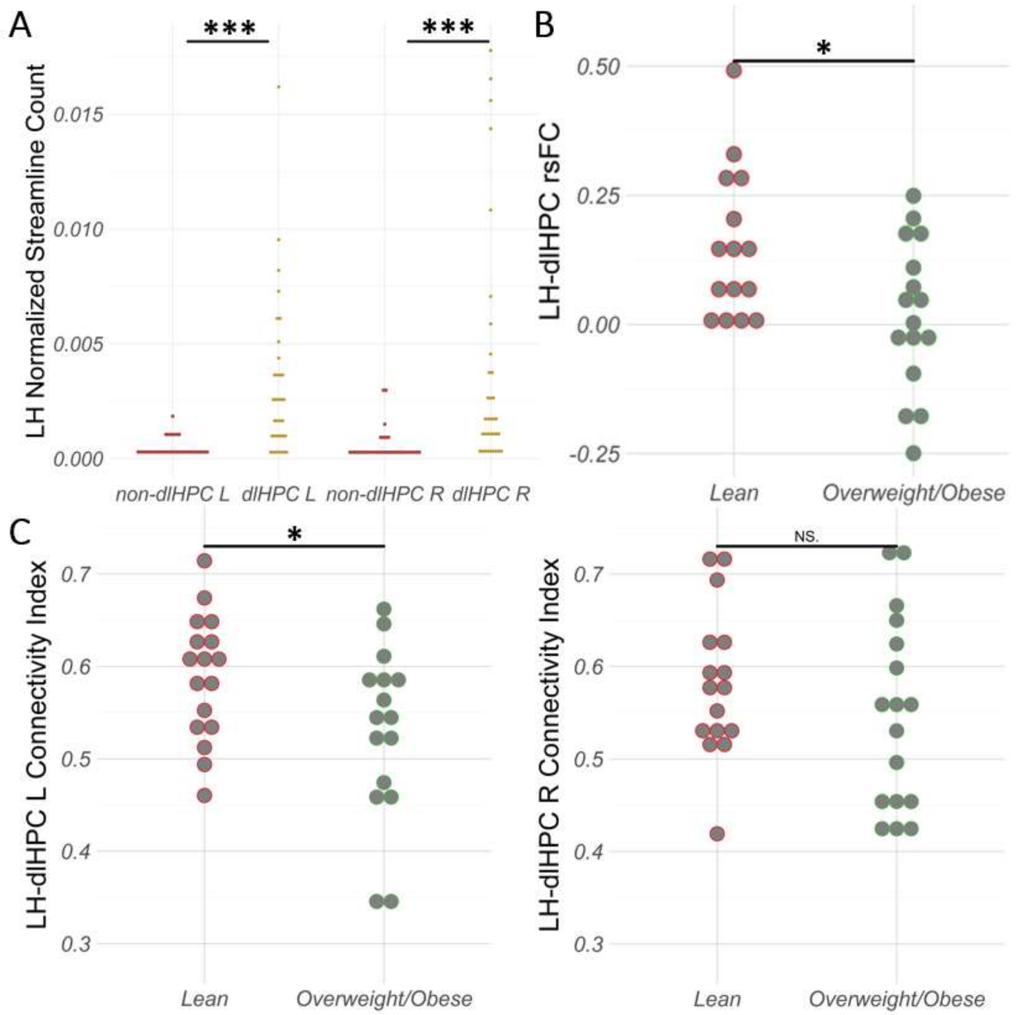

A
***
***
LH Normalized Streamline Count
0.015
0.010
0.005
0.000
non-dlHPC L
dlHPC L
non-dlHPC R
dlHPC R
B
*
LH-dlHPC rsFC
0.50
0.25
0.00
-0.25
Lean
Overweight/Obese
C
*
LH-dlHPC L Connectivity Index
0.7
0.6
0.5
0.4
0.3
Lean
Overweight/Obese
NS.
LH-dlHPC R Connectivity Index
0.7
0.6
0.5
0.4
0.3
Lean
Overweight/Obese
