# Supplementary material for: An orexigenic subnetwork within the human hippocampus
Source: Nature. 2023 Aug 30;621(7978):381–8. doi: 10.1038/s41586-023-06459-w (PMC10499606; doi:10.1038/s41586-023-06459-w)
Supplement: Supplementary file 2 — Reporting Summary [file 41586_2023_6459_MOESM2_ESM.pdf]

nature portfolio

Corresponding author(s): Casey H. Halpern

Last updated by author(s): Feb 21, 2023

Reporting Summary

Nature Portfolio wishes to improve the reproducibility of the work that we publish. This form provides structure for consistency and transparency in reporting. For further information on Nature Portfolio policies, see our Editorial Policies and the Editorial Policy Checklist.

Statistics

For all statistical analyses, confirm that the following items are present in the figure legend, table legend, main text, or Methods section.

n/a	Confirmed	
☐	☒	The exact sample size (*n*) for each experimental group/condition, given as a discrete number and unit of measurement
☐	☒	A statement on whether measurements were taken from distinct samples or whether the same sample was measured repeatedly
☐	☒	The statistical test(s) used AND whether they are one- or two-sided<br>*Only common tests should be described solely by name; describe more complex techniques in the Methods section.*
☐	☒	A description of all covariates tested
☐	☒	A description of any assumptions or corrections, such as tests of normality and adjustment for multiple comparisons
☐	☒	A full description of the statistical parameters including central tendency (e.g. means) or other basic estimates (e.g. regression coefficient) AND variation (e.g. standard deviation) or associated estimates of uncertainty (e.g. confidence intervals)
☐	☒	For null hypothesis testing, the test statistic (e.g. *F*, *t*, *r*) with confidence intervals, effect sizes, degrees of freedom and *P* value noted<br>*Give P values as exact values whenever suitable.*
☒	☐	For Bayesian analysis, information on the choice of priors and Markov chain Monte Carlo settings
☒	☐	For hierarchical and complex designs, identification of the appropriate level for tests and full reporting of outcomes
☒	☐	Estimates of effect sizes (e.g. Cohen's *d*, Pearson's *r*), indicating how they were calculated

Our web collection on statistics for biologists contains articles on many of the points above.

Software and code

Policy information about availability of computer code

Data collection	Electrophysiologic data was obtained from intracranially placed depth electrodes (AdTech Medical) by using the clinically available Nihon Kohden EEG recording system. Neuroimaging data from the binge-prone cohort was obtained with a 3T MRI scanner (Discovery MR750, GE Healthcare). 3D histology data was acquired using a UltraMicroscope II (Miltenyi BioTec, Germany).
Data analysis	Matlab Version 2020b, Python Version 3.6, FSL Version 6.0, Advanced Normalization Tools Version 2.1.0, fMRIPrep Version 1.2.3, DPABI/DPARSF Version 4.3, SPM Version 12 were used for the electrophysiologic and neuroimaging analyses in this study. Imaris Version 8.4 was used to render the histological images. Statistical analyses of the neuroimaging results were performed using the RStudio Version 1.2.5042. The code and materials used for analyzing the data in this study are available from the corresponding author upon reasonable request.

For manuscripts utilizing custom algorithms or software that are central to the research but not yet described in published literature, software must be made available to editors and reviewers. We strongly encourage code deposition in a community repository (e.g. GitHub). See the Nature Portfolio guidelines for submitting code & software for further information.

Data

Policy information about availability of data

All manuscripts must include a data availability statement. This statement should provide the following information, where applicable:

- Accession codes, unique identifiers, or web links for publicly available datasets
- A description of any restrictions on data availability
- For clinical datasets or third party data, please ensure that the statement adheres to our policy

Neuroimaging data were obtained from the publicly available Human Connectome Project 7T S1200 WashU-Minn-Ox HCP dataset (available at http://db.humanconnectome.org/). Electrophysiologic data were obtained from intracranially placed depth electrodes (AdTech Medical) by using the clinically available

Nihon Kohden EEG recording system. 3D histology data were acquired using a UltraMicroscope II (Miltenyi BioTec, Germany). Neuroimaging data from the binge-prone cohort were obtained with a 3T MRI scanner (Discovery MR750, GE Healthcare). Anonymized data that support the findings of this study are available from the corresponding author upon reasonable request.

Field-specific reporting

Please select the one below that is the best fit for your research. If you are not sure, read the appropriate sections before making your selection.

☐ Life sciences ☒ Behavioural & social sciences ☐ Ecological, evolutionary & environmental sciences

For a reference copy of the document with all sections, see nature.com/documents/nr-reporting-summary-flat.pdf

Behavioural & social sciences study design

All studies must disclose on these points even when the disclosure is negative.

Study description	The study with intracranial electrophysiology was a quantitative prospective study where we enrolled 9 consecutive patients with intracranial depth electrodes targeting the hippocampus placed for sole purposes of clinical monitoring. These patients were asked to participate in a cognitive task involving viewing a food cue and subsequently receiving a taste of either taste-neutral or palatable solution. The neuroimaging study enrolled 37 consecutive subjects (all female) with a history of weekly binge eating who expressed interest in participating in the imaging protocol.
Research sample	Normative data from all 178 unrelated subjects from the Human Connectome Project who underwent a ultra-high-resolution acquisition on a ''Magnetom'' 7T MRI scanner (Siemens Medical Systems, Erlangen, Germany) were obtained from the publicly available S1200 WashU-Minn-Ox HCP dataset. 9 consecutive human participants (2 female) with coverage of the hippocampus were enrolled for the intracranial electrophysiologic study. The baseline demographics are included in Table S1. 37 consecutive human participants (all female) with a history of binge eating behavior were enrolled for the imaging study.
Sampling strategy	Consecutive enrollment of patients meeting the enrollment criteria (for electrophysiology study: intracranial electrode targeting the hippocampus; for imaging study: history of weekly binge eating episodes). The electrophysiology study sample size was chosen to allow for adequate coverage of the hippocampus as well as based on clinical restraints; the imaging study sample size was limited by recruiting restraints imposed by the Covid-19 pandemic. We have included all possible subjects meeting inclusion criteria since the inception of the study.
Data collection	Nihon Kohden EEG-1200 and its supplied software was used for all video and EEG data capture in this study. No blinding was performed in this study. The researchers instructed the participants on the tasks and operated the electrophysiology hardware and software. For the neuroimaging data collection, we used a 3T MRI scanner (Discovery MR750, GE Healthcare, Milwaukee, Wisconsin).
Timing	The subject enrollment period (combined imaging and electrophysiology) was from September 22, 2015, to August 31, 2020
Data exclusions	No subject was excluded from electrophysiology study as subjects with lesions in the brain including mass, infarct and encephalomalacia were not enrolled. Three (out of 37) subjects were excluded from binge-prone imaging analysis due to excessive movement as measured by 1) mean framewise displacement (FD) > 0.2mm, 2) more than 20% of FD over 0.2mm, or 3) any FD > 5mm.
Non-participation	No enrolled subjects declined to participant.
Randomization	No randomization process apply to this study as there was no intervention. All subjects participated in the study tasks.

Reporting for specific materials, systems and methods

We require information from authors about some types of materials, experimental systems and methods used in many studies. Here, indicate whether each material, system or method listed is relevant to your study. If you are not sure if a list item applies to your research, read the appropriate section before selecting a response.

Materials & experimental systems

n/a	Involved in the study	
☐	☒	Antibodies
☒	☐	Eukaryotic cell lines
☒	☐	Palaeontology and archaeology
☒	☐	Animals and other organisms
☐	☒	Human research participants
☒	☐	Clinical data
☒	☐	Dual use research of concern

Methods

n/a	Involved in the study	
☒	☐	ChIP-seq
☒	☐	Flow cytometry
☐	☒	MRI-based neuroimaging

Antibodies

Antibodies used	Anti-melanin concentrating hormone. Phoenix Pharmaceuticals Inc, USA, H070-47, Lot No. 01629-10; Donkey anti-Rabbit Alexa Fluor 647 Plus, ThermoFisher Scientific, USA, A32795, Lot No. TF271041.
Validation	The primary antibody was validated for human immunohistochemistry and we confirmed that it was compatible with the reagents used in the immunolabeling-enabled 3D imaging of solvent cleared organs (iDISCO) protocol using the published iDISCO antibody validation protocol.

Human research participants

Policy information about studies involving human research participants

Population characteristics	See above.
Recruitment	Consecutive enrollment of patients meeting the enrollment criteria (for electrophysiology study: intracranial electrode targeting the hippocampus; for imaging study: history of weekly binge eating episodes). The electrophysiology study sample size was chosen to allow for adequate coverage of the hippocampus as well as based on clinical restraints; the imaging study sample size was limited by recruiting restraints imposed by the Covid-19 pandemic. We have included all possible subjects meeting inclusion criteria since the inception of the study.
Ethics oversight	The Stanford University Institutional Review Board approved the studies with the human electrophysiology, binge-prone cohorts, and the human hippocampal sample.

Note that full information on the approval of the study protocol must also be provided in the manuscript.

Magnetic resonance imaging

Experimental design

Design type	N/A - the MRI portion of this study did not involve a task or stimulus-based design.
Design specifications	N/A - the MRI portion of this study did not involve a task or stimulus-based design.
Behavioral performance measures	N/A - the MRI portion of this study did not involve measures of behavioral performance.

Acquisition

Imaging type(s)	Diffusion, functional and structural
Field strength	7T and 3T
Sequence & imaging parameters	Different sequences and parameters apply for the two different imaging datasets used. They were described in detail in Table S2.
Area of acquisition	Brain

Diffusion MRI ☒ Used ☐ Not used

Parameters	130 directions (normative data) and 37 directions (binge eating data), multi-shell (normative data) and single-shell, no cardiac gating was used.

Preprocessing

Preprocessing software	Diffusion MRI data was preprocessed using the FSL suite. Functional MRI data was preprocessed using fMRIPrep 1.2.3 (6mm FWHM isotropic Gaussian kernel, ICA-AROMA was used to identify motion-related noise components in the BOLD signal).
Normalization	Advanced Normalization Tools (ANTs) was used for non-linear transformation of ROIs to native space of research participants.
Normalization template	Individual segmentation results were normalized to the MNI152 09c template for population average definition.
Noise and artifact removal	For individual measurements, data the analysis was conducted in native space. For group level results (hippocampal segmentation), data were normalized to MNI152 09c template.
Volume censoring	For functional MRI data, non-steady state volumes were censored by fMRIPrep 1.2.3

Statistical modeling & inference

Model type and settings	N/A - this study did not involve task or stimulus-based functional MRI.

Effect(s) tested	N/A - this study did not involve task or stimulus-based functional MRI.

Specify type of analysis: ☐ Whole brain ☒ ROI-based ☐ Both

Anatomical location(s)	The lateral hypothalamus (LH) region mask was defined on the standard T1 MNI152 09c template adapted from CIT168 Subcortical In Vivo Probabilistic Atlas, while the hippocampi masks were defined using the Harvard-Oxford brain atlas. This registration was performed using Advanced Normalization Tools (ANTs), which consists of two successive steps of linear and nonlinear registration between the subject‟s brain and the MNI brain. In a third step, the MNI-defined ROIs were registered to the subject's space.
Statistic type for inference (See Eklund et al. 2016)	ROI-based.
Correction	No correction for multiple comparisons were applicable since the hypothesis-driven analysis involved a single pair of ROIs.

Models & analysis

n/a	Involved in the study
☐	☒ Functional and/or effective connectivity
☒	☐ Graph analysis
☐	☒ Multivariate modeling or predictive analysis

Functional and/or effective connectivity	Functional connectivity measurements where calculated using DPABI 4.3/DPARSF which is based on Statistical Parametric Mapping (SPM12).
Multivariate modeling and predictive analysis	A multivariate logistic regression model was used to predict whether a subject belongs to the overweight/obese or lean group including the available demographic and behavioral variables in addition to the LH-dlHPC connectivity measurements. The comprehensive list of variables included: age, depression (BDI), anxiety, binge frequency, restrained eating, emotional eating and externally driven eating scores (from DEBQ), LH-dlHPC-LH rsFC, LH-non-dlHPC rsFC, LH-dlHPC structural connectivity, and LH-non-dlHPC structural connectivity. We then used backwards elimination to identify which combination of variables provided highest predictive power with the lowest total number of explanatory variables to avoid over-fitting (Akaike Information Criterion).
