# Supplementary material for: An orexigenic subnetwork within the human hippocampus
Source: Nature. 2023 Aug 30;621(7978):381–8. doi: 10.1038/s41586-023-06459-w (PMC10499606; doi:10.1038/s41586-023-06459-w)
Supplement: Supplementary file 3 — Supplementary Tables 1–4. [file 41586_2023_6459_MOESM3_ESM.docx]

**SI Table 1: Participant characteristics.**

| **ID** | **Age** | **Sex** | **Handedness** | **(Side) Seizure Focus** | **BMI** | **Sweet-fat Solution Rating** | **Preferred Sweet-fat over Taste-neutral?** | **Number of Contacts (side)** | |
| --- | --- | --- | --- | --- | --- | --- | --- | --- | --- |
|  |  |  |  |  |  |  |  | **dlHPC** | **Non-dlHPC** |
| **S1** | 59 | M | R | Bitemporal | 26 | 5/10 | Yes | 2(L) 2(R) | 3(L) 2(R) |
| **S2** | 34 | F | R | (L) Lateral Temporal | 32 | 3/10 | Yes | 2(L) | 1(L) 2(R) |
| **S3** | 24 | M | R | (R) Mesial Temporal | 51 | 3/10 | Yes | 4(R) | 0 |
| **S4** | 49 | M | R | (L) Frontal and L. Mes. Temporal | 31 | 8/10 | Yes | 1(L) | 2(L) |
| **S5** | 51 | M | R | (L) Mesial Temporal | 27 | 10/10 | Yes | 2(L) 6(R) | 1 (L) 2 (R) |
| **S6** | 20 | M | R | (R) Parietal/Occipital | 19 | 5/10 | Yes | 2(L) | 0 |
| **S7** | 27 | M | R | (R) Parietal | 24 | 8/10 | Yes | 1(L) | 1(L) |
| **S8** | 36 | M | R | Bitemporal | 23 | 7/10 | Yes | 2(L) 4(R) | 4(L) |
| **S9** | 29 | F | R | (R) Frontal Dysplasia | 41 | 3/10 | Yes | 5(L) 4(R) | 1(L) 1(R) |

**SI Table 2: Summary of MRI acquisition parameters.**

|  | **HCP Dataset** | **Binge-eating Cohort** | | |
| --- | --- | --- | --- | --- |
| **Data Type** | Diffusion | Diffusion | Structural (T1w) | Resting-state |
| **Magnetic Field** | 7T | 3T | 3T | 3T |
| **Sequence** | Spin-Echo EPI | Spin-Echo EPI | BRAVO | SPIRAL |
| **TE / TR (ms)** | 71.2 / 7000 | 97.5 / 12800 | 1.87 / 5.8 | 30 / 2000 |
| **Flip Angle (**°) | 90 | 90 | 11 | 80 |
| **Field-of-View (mm²)** | 210 | 220 | 230 | 220 |
| **Matrix Size** | 200 (acquisition) x 200 (recon) | 128 (acquisition) x 256 (recon) | 192 (acquisition) x 256 (recon) | 128 (acquisition) x 64 (recon) |
| **In-plane Res. (mm²)** | 1.05x1.05 | 0.8594x0.8594 | 0.9375x0.9375 | 3.4x3.4 |
| **Slice Thickness (mm)** | 1.05 | 2.3 | 1.2 | 3.5 |
| **Number of Slices** | 132 Axial | 74 Axial | 146 Axial | 31 Axial |
| **Runs** | 4 | 1 | 1 | 1 |
| **Acquisition Time (min)** | 9:50 (39:20 total) | 15:23 | 8:40 | 8:00 |
| **Diffusion Encoding (s/mm²)** | 6 × b=0  65 dir. × b=1000,  65 dir. × b=2000 | 1 × b=0  37 dir. × b=1000 | N/A | N/A |

**SI Table 3: Summary of demographics, clinical and behavioral assessments across our full cohort as well as within the lean and overweight/obese groups separately.** SD = standard deviation; BDI = Beck’s depression inventory; BAI = Beck’s anxiety inventory; DEBQ = Dutch eating behavior questionnaire. *Student’s T test (two-sided) comparing lean and obese groups.

|  | **Binge-prone cohort** | | |  |  |
| --- | --- | --- | --- | --- | --- |
|  | **All**  **n = 34** | **Lean (BMI < 25)**  **n = 17** | **Overweight/Obese (BMI > 35) n = 17** | **Student’s T** | ***p**** |
| Age in years (SD) | 27 (6) | 26 (6) | 27 (6) | –0.84 | .41 |
| BMI (SD) | 27.7 (8) | 21.9 (1.8) | 33.6 (7.6) | **-** | **-** |
| Binge frequency per week (SD) | 2.7 (1.4) | 2.4 (1.2) | 2.9 (1.6) | –0.92 | .36 |
| Purging frequency per week (SD) | 0.2 (0.5) | 0.4 (0.7) | 0 (0.1) | 2.03 | .06 |
| BDI-I (SD) | 13 (8.1) | 10.6 (8.1) | 15.5 (7.4) | –1.84 | .08 |
| BAI (SD) | 10.1 (6.4) | 11 (6.7) | 9.2 (6.2) | 0.82 | .42 |
| DEBQ restrained eating (SD) | 3.2 (0.8) | 3.1 (0.7) | 3.2 (1) | –0.31 | .76 |
| DEBQ emotional eating (SD) | 3.6 (1) | 3.5 (1.1) | 3.7 (1) | –0.44 | .66 |
| DEBQ externally driven eating (SD) | 3.5 (0.7) | 3.5 (0.7) | 3.5 (0.6) | –0.13 | .90 |

**SI Table 4: Summary of rsFC between the LH and control regions in our full cohort as well as within the lean and** **overweight/obese groups separately.** LH = Lateral hypothalamus; rsFC = resting-state functional connectivity; SD = standard deviation. *Student’s T test (two-sided) comparing lean and overweight/obese groups.

|  | **Binge-prone cohort** | | |  |  |
| --- | --- | --- | --- | --- | --- |
|  | **All** | **Lean (BMI < 25)** | **Overweight/Obese (BMI > 35)** | **Student’s T** | ***p**** |
| LH-amygdala rsFC (SD) | 0.23 (0.21) | 0.23 (0.18) | 0.22 (0.23) | 0.12 | .91 |
| LH-hippocampus rsFC (SD) | 0.12 (0.20) | 0.16 (0.23) | 0.08 (0.18) | 1.20 | .24 |
| LH-motor cortex rsFC (SD) | 0.00 (0.15) | 0.02 (0.15) | –0.01 (0.15) | 0.50 | .62 |
